# Towards the understanding of the enzymatic cleavage of polyisoprene by the dihaem-dioxygenase RoxA

**DOI:** 10.1186/s13568-019-0888-0

**Published:** 2019-10-17

**Authors:** Georg Schmitt, Jakob Birke, Dieter Jendrossek

**Affiliations:** 1 Institute of Microbiology, University of Stuttgart, Allmandring 31, 70569 Stuttgart, Germany; 2 Present Address: Institute of Applied Biotechnology, University of Applied Sciences Biberach, Hubertus-Liebrecht-Strasse 35, 88400 Biberach, Germany

**Keywords:** Rubber oxygenase, RoxA, Haem, Dioxygenase, Polyisoprene, Rubber biodegradation

## Abstract

Utilization of polyisoprene (natural rubber) as a carbon source by *Steroidobacter cummioxidans* 35Y (previously *Xanthomonas* sp. strain 35Y) depends on the formation and secretion of rubber oxygenase A (RoxA). RoxA is a dioxygenase that cleaves polyisoprene to 12-oxo-4,8-dimethyl-trideca-4,8-diene-1-al (ODTD), a suitable growth substrate for *S. cummioxidans*. RoxA harbours two non-equivalent, spectroscopically distinguishable haem centres. A dioxygen molecule is bound to the N-terminal haem of RoxA and identifies this haem as the active site. In this study, we provide insights into the nature of this unusually stable dioxygen-haem coordination of RoxA by a re-evaluation of previously published together with newly obtained biophysical data on the cleavage of polyisoprene by RoxA. In combination with the meanwhile available structure of RoxA we are now able to explain several uncommon and previously not fully understood features of RoxA, the prototype of rubber oxygenases in Gram-negative rubber-degrading bacteria.

## Introduction

The hydrocarbon natural rubber [poly(*cis*-1,4-isoprene)] is used in huge amounts for the production of tires and countless other items in industry and private households since decades. The majority of the items are released to the environment after use. Rubber-degrading bacteria are present in most ecosystems and contribute to a substantial biodegradation of these materials. In particular, small rubber particles originating from tires by abrasion do not visibly accumulate in the environment, indicating the efficient biodegradation of polyisoprene-containing materials. Research on the biochemical and molecular biological mechanisms of polyisoprene biodegradation has been performed in the last two decades by several groups. The utilization of polyisoprene as a carbon source depends on the ability to cleave the hydrocarbon polymer extracellularly to low molecular products that are small enough to permeate the cell membrane (Jendrossek et al. 1997; Rose and Steinbüchel 2005; Yikmis and Steinbüchel 2012a; Jendrossek and Birke 2018). Rubber oxygenases catalysing the initial cleavage of polyisoprene were evolved separately in Gram-positive and Gram-negative rubber utilizing bacteria and rely on latex clearing protein (Lcp) or on rubber oxygenase A and rubber oxygenase B (RoxA, RoxB), respectively (Jendrossek and Reinhardt 2003; Braaz et al. 2004; Rose et al. 2005; Yikmis and Steinbüchel 2012b; Birke et al. 2017). Lcps have been purified and biochemically characterised from *Gordonia polyisoprenivorans* VH2 (Hiessl et al. 2014; Oetermann et al. 2018), *Streptomyces* sp. K30 (Birke and Jendrossek 2014; Birke et al. 2015), *Rhodococcus rhodochrous* RPK1 (Watcharakul et al. 2016) and from a *Nocardia* sp. strain (Linh et al. 2017). Lcps are ≈ 40 kDa proteins that share a common domain of unknown function (DUF2236) and harbour one non-covalently bound *b*-type haem group as cofactor (Hiessl et al. 2014; Birke et al. 2015; Röther et al. 2016; Oetermann et al. 2018). Lcps oxidatively cleave poly(*cis*-1,4-isoprene) at the double bounds to a mixture of low molecular products (C_20_, C_25_, C_30_ and higher oligoisoprenoids) all which have the same terminal functions, CHO–CH_2_– and –CH_2_–COCH_3_ but differ in the number of intact isoprene units in between (Ibrahim et al. 2006; Birke and Jendrossek 2014; Röther et al. 2017a). Recently, the 3-D structure of Lcp was solved, revealing a protein core with a classical 3/3 globin fold and an oxidised (ferric) haem iron that possesses histidine (H198) and lysine (K167) as axial ligands (Ilcu et al. 2017). The ability to degrade and utilise rubber as a carbon source is far less distributed among Gram-negative bacteria and only a few species of the *beta*-, *gamma*- and *delta*-*proteobacteria* have been identified (Tsuchii and Takeda 1990; Imai et al. 2013; Birke et al. 2013). The best studied strain is *S. cummioxidans* 35Y (previously *Xanthomonas* sp. 35Y, (Tsuchii and Takeda 1990; Sharma et al. 2018) that harbours rubber oxygenase A (RoxA), the first isolated and biochemically characterised rubber oxygenase (Braaz et al. 2004, 2005; Schmitt et al. 2010). RoxA is a ≈ 70 kDa protein with two covalently attached haem cofactors which identify it as a *c*-type cytochrome. The RoxA 3-D structure revealed a structural relationship to bacterial cytochrome *c* peroxidases (CCPs) (Seidel et al. 2013). Despite this, RoxA does not feature a peroxidase activity. RoxA cleaves natural rubber in a processive manner to the C_15_ oligoisoprenoid 12-oxo-4,8-dimethyl-trideca-4,8-diene-1-al (ODTD) as major end product. The N-terminal haem group was identified as the active site of the enzyme. The axial ligands of this haem group consist of His195 and, surprisingly, a firmly bound dioxygen molecule (Seidel et al. 2013). The coordination of dioxygen is well known for globins such as haemoglobin or myoglobin but is unique among haem containing (di)oxygenases, possibly due to the high reactivity of the bound dioxygen molecule that can lead to destruction of the porphyrin or the protein.

Very recently, a third type of rubber dioxygenase, RoxB, and the corresponding homologs were discovered in *S. cummioxidans* 35Y and all other currently known Gram-negative rubber degrading bacteria (Kasai et al. 2017; Birke et al. 2017; Röther et al. 2017a). RoxB_Scu_ is moderately related to RoxA_Scu_ in amino acid sequence and also harbours two covalently bound haem molecules. In contrast to those similarities, RoxB yielded a mixture of oligoisoprenoid cleavage products largely differing in the number of isosprene units, which is in sharp contrast to the RoxA catalysed reaction that leads to the formation of only one (major) end product, ODTD. Since RoxA and RoxB were expressed simultaneously in the wild type strain, a cooperative effect on the rubber degradation was suggested and experimentally confirmed (Birke et al. 2017; Röther et al. 2017a). For a recent overview on the properties of purified and biochemically characterised rubber oxygenases and rubber oxygenase assay methods see (Röther et al. 2017b; Jendrossek and Birke 2018).

The presence of haem groups is causative for the red or red-brownish colour of concentrated solutions of rubber oxygenases. Since even minor changes in the spatial orientation of haem ligands or of the haem environment cause specific alterations in the optical spectra of the proteins in the range between 200 and 700 nm (hereafter designated as UV–vis spectra) UV–vis spectroscopy is a suited tool for studying these enzymes. In the past, RoxA-wild type (Wt) and RoxA-muteins with site-specific exchanges of selected amino acid residues have been investigated by UV–vis and electron paramagnetic resonance (EPR) spectroscopy (Schmitt et al. 2010; Birke et al. 2012). Since some of the previously made observations were unusual and not fully understood, it is the aim of this study to re-evaluate these findings and to combine our data with newly obtained spectroscopic and biochemical insights to provide a more comprehensive picture of the RoxA-catalysed cleavage of polyisoprene.

## Materials and methods

### Bacterial strains, plasmids and culture conditions

Table 1 shows the bacterial strains and plasmids that were used in this study. Recombinant *E. coli* strains were grown in lysogeny broth (LB) medium at 37 °C in the presence of the appropriate antibiotic (kanamycin or ampicillin). The expression strain *S. cummioxidans* 35Y, harbouring *roxA*(-variant) gene of interest, was grown for 72 h at 23 °C in modified LB medium (per liter: 5 g NaCl, 0.3 g yeast extract, 10 g tryptone) that had been supplemented with 0.1% (wt/vol) of l-rhamnose as inducer as described in detail elsewhere (Birke et al. 2012). Polyisoprene latex was used after 3 washing steps in 0.1% (wt/vol) Nonidet P40 to remove stabilizing compounds [provided by Weber and Schaer, Hamburg (Germany)]. The preparation of latex overlay agar in mineral salts medium [Tsuchii and Takeda medium (1990) supplemented with 0.1% yeast extract] has been described previously (Birke et al. 2012).

**Table 1:** Bacterial strains and plasmids used in this study

Strain/plasmid	Relevant characteristics	References
*Escherichia coli* S17-1	Conjugation strain	Simon et al. (1983)
*Steroidobacter cummioxidans* 35Y	Growth on poly(*cis*-1,4-isoprene) latex, clearing zone formation	DSMZ103114
*S. cummioxidans* 35Y-CM	Chloramphenicol resistant mutant of 35Y	Hambsch et al. (2010)
*S. cummioxidans* 35Y-CM Δ*roxA*-*attB* (SN3727)	Chromosomal deletion of *roxA*, *attB* at former *roxA* site no clearing zone formation on latex agar	Birke et al. (2012)
*S. cummioxidans* 35Y-CM Δ*roxA*-*attB* pNH1-*roxA*-*attP* (SN4230)	Expression of *roxA* from rhamnose promotor Km^r^, Cm^r^, clearing zone formation in the presence of rhamnose	Birke et al. (2012)
*S. cummioxidans* 35Y-CM Δ*roxA(F317Y)*-*attB* pNH1-*roxA*-*attP*	Expression of *roxA* (F317Y) from rhamnose promotor, Km^r^, Cm^r^, clearing zone formation in the presence of rhamnose	Birke et al. (2012)
*S. cummioxidans* 35Y-CM Δ*roxA(F317L)*-*attB* pNH1-*roxA*-*attP*	Expression of *roxA* (F317L) from rhamnose promotor, Km^r^, Cm^r^, clearing zone formation in the presence of rhamnose	Birke et al. (2012)
*S. cummioxidans* 35Y-CM Δ*roxA(F317A)*-*attB* pNH1-*roxA*-*attP*	Expression of *roxA* (F317A) from rhamnose promotor, Km^r^, Cm^r^, clearing zone formation in the presence of rhamnose	Birke et al. (2012)
*S. cummioxidans* 35Y-CM Δ*roxA(F301L)*-*attB* pNH1-*roxA*-*attP*	Expression of *roxA* (F317L) from rhamnose promotor, Km^r^, Cm^r^, clearing zone formation in the presence of rhamnose	This study
*S. cummioxidans* 35Y-CM Δ*roxA(F301Y)*-*attB* pNH1-*roxA*-*attP*	Expression of *roxA* (F317Y) from rhamnose promotor, Km^r^, Cm^r^, clearing zone formation in the presence of rhamnose	This study

### Cloning of *roxA*(-variants)

Cloning of *roxA*-(variants) was described in detail previously (Birke et al. 2012). In brief, the *roxA* gene was modified by QuikChange PCR, pUC9::*roxA* was used as template. The gene was cloned in the expression vector pNH1. The resulting plasmid pNH1::*roxA*-variant was conjugatively transferred from *E. coli* S17-1 to *S. cummioxidans* 35Y Δ*roxA* and chromosomally integrated via *attP/attB* recombination. The chromosomal integration of the expression vector as well as the *roxA*-sequence was confirmed by colony-PCR and subsequent DNA sequencing.

### Purification of RoxA(-variants)

Purifications were performed as described previously (Schmitt et al. 2010; Birke et al. 2012). RoxA was purified from the supernatant of a *S.* *cummioxidans* 35Y-*roxA*-*attB* pNH1::*roxA*(-variant) culture that was grown in 12 individual 600-ml cultures of modified LB medium (each in a 3-l Erlenmeyer flask), supplemented with 0.1% (wt/vol) l-rhamnose for 72 h at 23 °C, 120 rpm. Cells were harvested by centrifugation (4 °C, 16,000*g*), the supernatant was concentrated by ultrafiltration (10-kDa cut-off) to a volume of 350 ml and applied to a Q-Sepharose fast-flow column (Q-FF 50/11, bed volume 250 ml) that had been equilibrated with 20 mM Tris–HCl (pH 8.5; flow rate 8 ml/min). RoxA was detected by following the absorbance at 407 nm and was eluted in a subsequent step gradient at ≈ 50 mM NaCl in equilibration buffer. Combined RoxA-containing fractions were concentrated to a volume of 50 ml (Amicon, 30 kDa cut-off), Tris–HCl buffer was exchanged by potassium phosphate buffer (10 mM, pH 6.8) by gel filtration with a HiPrep 26/10 desalting column (GE-healthcare, UK). Subsequently, the RoxA pool was applied to a hydroxyapatite column (CHT5-I, bed volume 20 ml) that had been equilibrated with the same buffer. RoxA was eluted with a linear gradient of 10 to 200 mM potassium phosphate buffer, pH 6.8. RoxA fractions were pooled and stored on ice or frozen in liquid nitrogen and stored at − 70 °C. Purity was tested by sodium dodecyl sulphate–polyacrylamide gel electrophoresis (SDS-PAGE) and by determination of the absorption quotient 407 nm/280 nm.

### Assay of rubber oxygenase activity

To determine the activity of RoxA, two different assays were applied: (i) the consumption of dissolved oxygen was determined in an OXY-4 mini apparatus (PreSens, Regensburg, Germany) as described previously (Birke and Jendrossek 2014; Röther et al. 2017b). This allows the determination of the specific activity as well as the relative activities of RoxA(-variants) by monitoring the consumption of the co-substrate dioxygen as a result of polyisoprene cleavage. (ii) for the second assay, polyisoprene latex was incubated in the presence of the test enzyme for 1 or 2 h at 23 °C or 30 °C. The cleavage products were extracted with ethyl-acetate and separated by HPLC as described previously (Birke and Jendrossek 2014). For determination of the relative activities of RoxA-variants, the peak area of the cleavage product peak (ODTD) with a retention time of ≈ 15.3 min was used.

### Other techniques

The concentration of protein solutions was determined by the bicinchoninic acid (BCA) method using a commercial BCA kit (Pierce). Separation of proteins was performed by polyacrylamide gel electrophoresis in the presence of sodium dodecyl sulphate (SDS-PAGE) under reducing (2-mercaptoethanol) conditions. SDS-PAGE gels were stained with silver (Blum et al. 1987). UV–vis and EPR spectroscopy were performed using purified RoxA as described previously (Schmitt et al. 2010; Birke et al. 2012; Seidel et al. 2013; Ilcu et al. 2017). The relative EPR-intensity is displayed. EPR spectra are shown after subtraction of the buffer signal, if not stated otherwise. However, for many spectra a complete removal of the background was not achieved, which especially affects the “radical region” around 340 mT.

## Results

### Dioxygen is stably bound to the catalytic haem centre of RoxA

The determination of the structure of RoxA from *S. cummioxidans* 35Y at high resolution revealed the presence of a dioxygen molecule bound to the N-terminal haem group thereby confirming that this haem represents the active site of the enzyme. EPR analysis of RoxA *as isolated* suggested that the iron atom of the active site haem is mainly present in an EPR-silent Fe^2+^–O_2_↔Fe^3+^–O_2_^−^ equilibrium. In this contribution the term “*as isolated*” is used to indicate a purified rubber oxygenase preparation that was obtained at conditions of an atmospheric gas phase and to which no compounds than those of the purification buffer had been added.

**Fig. 1:**
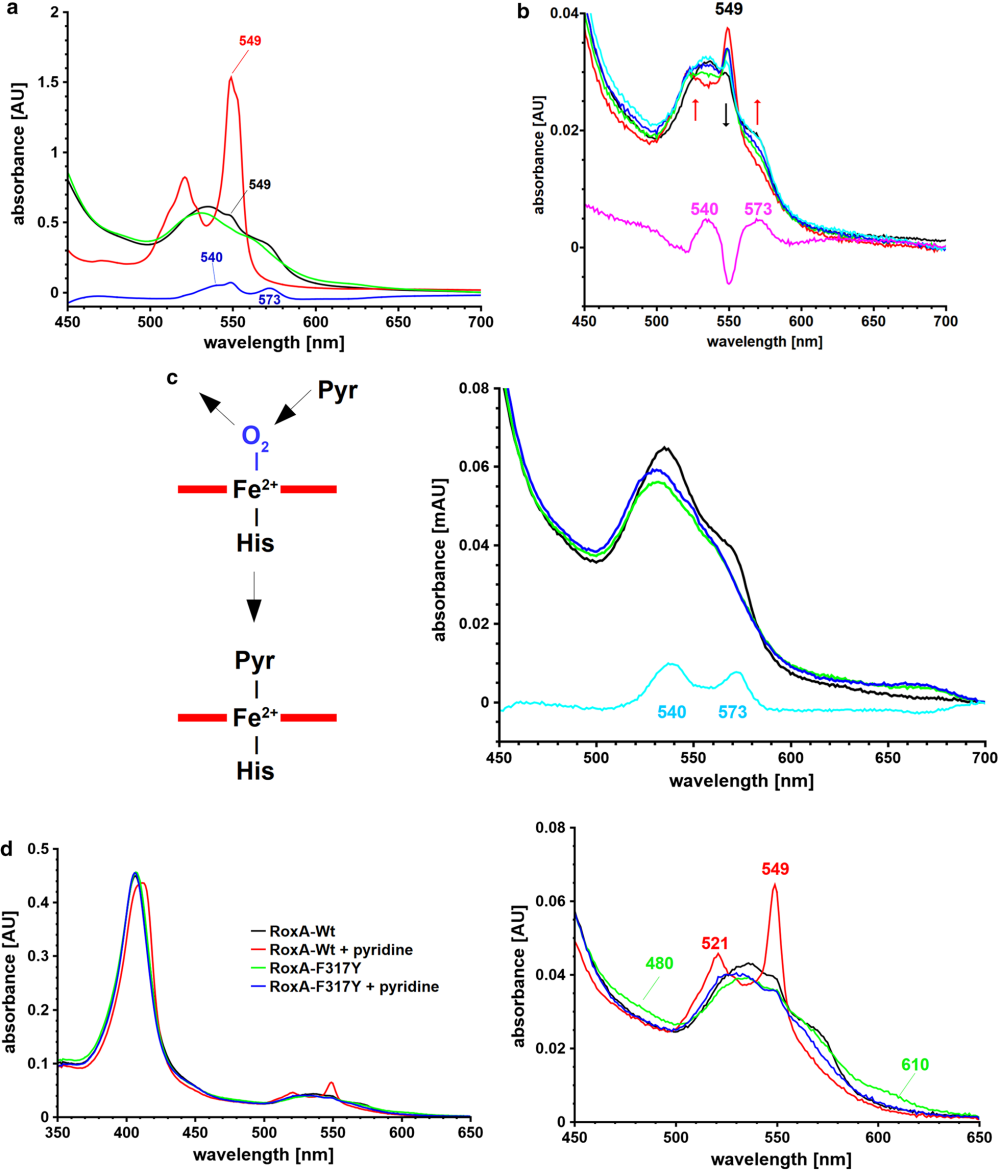

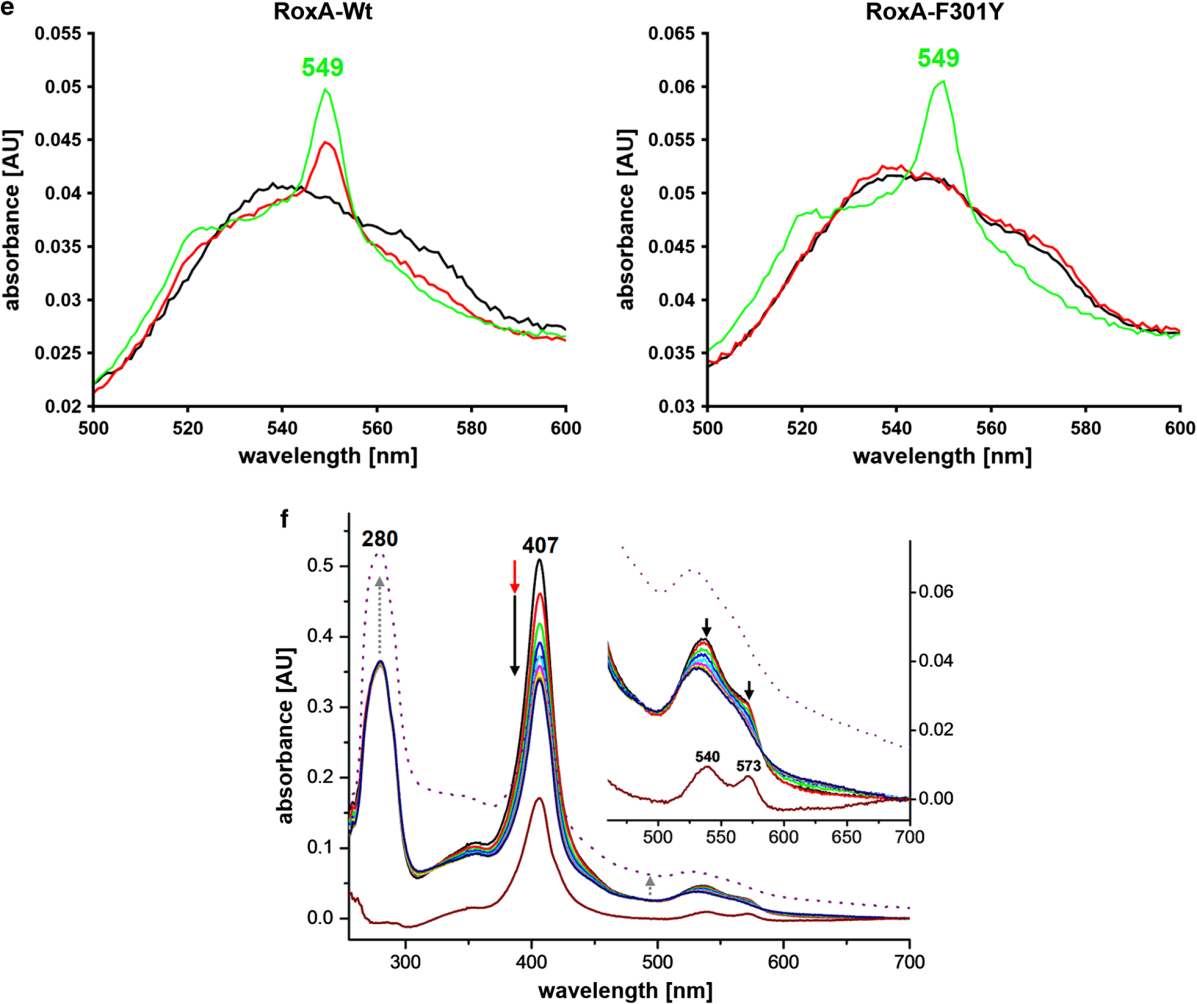
UV–vis spectra **a** Q-band region of RoxA *as isolated* (black), dithionite reduced (2 mM, red), dithionite reduced and subsequently ferricyanide-oxidised (10 mM, green) and a difference spectrum (*as isolated* minus ferricyanide-oxidised: blue). The difference spectrum shows features at 540 nm and 573 nm after ferricyanide reoxidation. This is characteristic for the loss of haem-bound dioxygen. The minor band at 549 nm in RoxA *as isolated* represents a small fraction of RoxA molecules with a (deoxygenated) reduced N-terminal haem centre. **b** Reoxidation of deoxygenated RoxA. UV–vis spectra (Q-band region) of RoxA after reduction with dithionite (twofold molar excess to haem, N_2_ atmosphere, red graph) and subsequent reoxidation under a pure O_2_-atmosphere. Arrows mark the direction of Q-band changes characteristic for the formation of oxygenated haem (red arrows) by concomitant decrease of reduced haem (black arrow). The green, dark blue, light blue and black lines indicate dithionite-reduced RoxA preparations that were exposed to dioxygen for 5 min, 1 h, 2 h or 4 h, respectively. The pink line shows the difference spectrum of the reaction (black minus red). The black graph after 4 h exposure to oxygen atmosphere basically represents the state *as isolated* which is oxygenated. For better visualization the spectrum of RoxA *as isolated* had been, however, omitted. Please note, that this experiment starts with the N-terminal haem in the reduced state and does not require a fully reduced enzyme as it is shown representatively in **a** (red). **c** Reaction of RoxA with pyridine. (left scheme): the haem-bound dioxygen molecule in the *as isolated* state of RoxA (Fe^2+^–O_2_) can be substituted by N-heterocyclic compounds, e.g. pyridine (Fe^2+^-Pyr) or imidazole. (right): UV–vis spectra of RoxA *as isolated* (black), ferricyanide-reoxidised (green), ferricyanide-reoxidised + pyridine-incubated (blue). In contrast to RoxA *as isolated*, only minor changes in the optical spectrum occur upon pyridine (imidazole) incubation for reoxidised RoxA. This can be explained by binding of pyridine (imidazole) to an oxidised, deoxygenated haem centre. The light blue line shows the difference spectrum of RoxA *as isolated* minus anaerobically reoxidised RoxA. **d** RoxA-Wt and RoxA-F317Y after incubation with pyridine. In RoxA-Wt, the “reduction bands” are caused by replacement of dioxygen (Fe^2+^–O_2_) with pyridine (Fe^2+^-pyridine) from the N-terminal haem centre. This state resembles the spectrum of reduced RoxA (Fe^2+^). In contrast, RoxA-F317Y *as isolated* primarily rests in the oxidised state (Fe^3+^). Thus, the binding of pyridine (Fe^3+^-pyridine) does not result in a reduced spectrum. **e** UV–vis spectroscopic changes of RoxA-Wt and RoxA-F301Y by imidazole or pyridine at room temperature. *As isolated* (black); incubation with imidazole (red); incubation with pyridine after imidazole incubation (green). In RoxA-Wt, the replacement of dioxygen by imidazole was finished within 100 min at RT, whereas RoxA-F301Y was not affected by imidazole. After addition of 2 mM pyridine, RoxA-F301Y slowly (20 min) converted to the reduced spectrum. For RoxA-Wt almost no additional changes were observed. The total increase of absorption at 549 nm is comparable for both proteins indicating that the portion of proteins with a bound dioxygen molecule is similar for RoxA-Wt and RoxA-F301Y. **f** Effect of H_2_O_2_ on the UV–vis spectrum of RoxA. UV–vis spectra of RoxA-Wt (2.5 µM) were recorded before (black line) and every 2 min after the addition of 1 mM H_2_O_2_. Black arrows indicate the time-dependent changes of haem absorption (every 2 min until endpoint of the haem-destructive effect, indicated by different colours), the red arrow shows the immediate decrease after addition of H_2_O_2_. Prolonged incubation after the end-point of haem destruction leads to an increase of the 280 nm absorption, but no further decrease of haem absorption indicating an oxidative destruction of the protein backbone (dotted line and arrows). The difference between RoxA-Wt *as isolated* and after reaction with H_2_O_2_ at the endpoint of haem-destructive reaction is illustrated on the bottom (brown line), reflecting the spectrum of the formerly oxygenated N-terminal haem centre

A stable binding of dioxygen to haem iron is well known for haemoglobin but has not been found in haem-dependent (di)oxygenases except for RoxA. A possible reason for this might be the chemical reactivity of dioxygen that can lead to a rapid destruction of the haem cofactor and of the protein (Batabyal and Yeh 2009; Seidel et al. 2013). The optical spectrum of RoxA in the *as isolated* state exhibited a decrease at 540 nm and 573 nm after exposure to ferricyanide or by addition of the O_2_-consuming agent pyrogallol (Additional file 1: Fig. S1). A similar effect was observed for dithionite-reduced and subsequently ferricyanide-oxidised RoxA (Fig. 1a). These spectroscopic features agree well with those of metmyoglobin [see Fig. 2 of Ghafourifar et al. (2005)]. They are characteristic for an oxidised haem group and indicate the loss of the formerly haem-bound dioxygen. A loss of the dioxygen molecule from the RoxA active site also explains previously published data, where under low oxygen gas pressure conditions (N_2_ atmosphere or vacuum) an increase of the 549 nm α-band was observed (Schmitt et al. 2010), Additional file 1: Fig. S2). These features support our assumption that the haem iron of the active site in RoxA *as isolated* is present in an Fe^2+^–O_2_↔Fe^3+^–O_2_^−^ equilibrium. If dioxygen is removed, the ferrous state of iron becomes detectable as a partially reduced UV–vis spectrum; amongst other characteristic spectral changes this can be seen as an increase of the 549 nm α-band. In this contribution, the increasing absorption band at 549 nm in an UV–vis spectrum of a RoxA preparation by removal of bound dioxygen or by replacement with small ligand molecules will be referred as “reduction band” although this does not correspond to a true reduction but to a fixation of the Fe^2+^–O_2_↔Fe^3+^–O_2_^−^ equilibrium to the ferrous Fe^2+^ state. The reversible binding of dioxygen in dependence of the partial oxygen gas pressure is well-known for dioxygen-transport proteins such as haemoglobin or myoglobin.

**Fig. 2:**
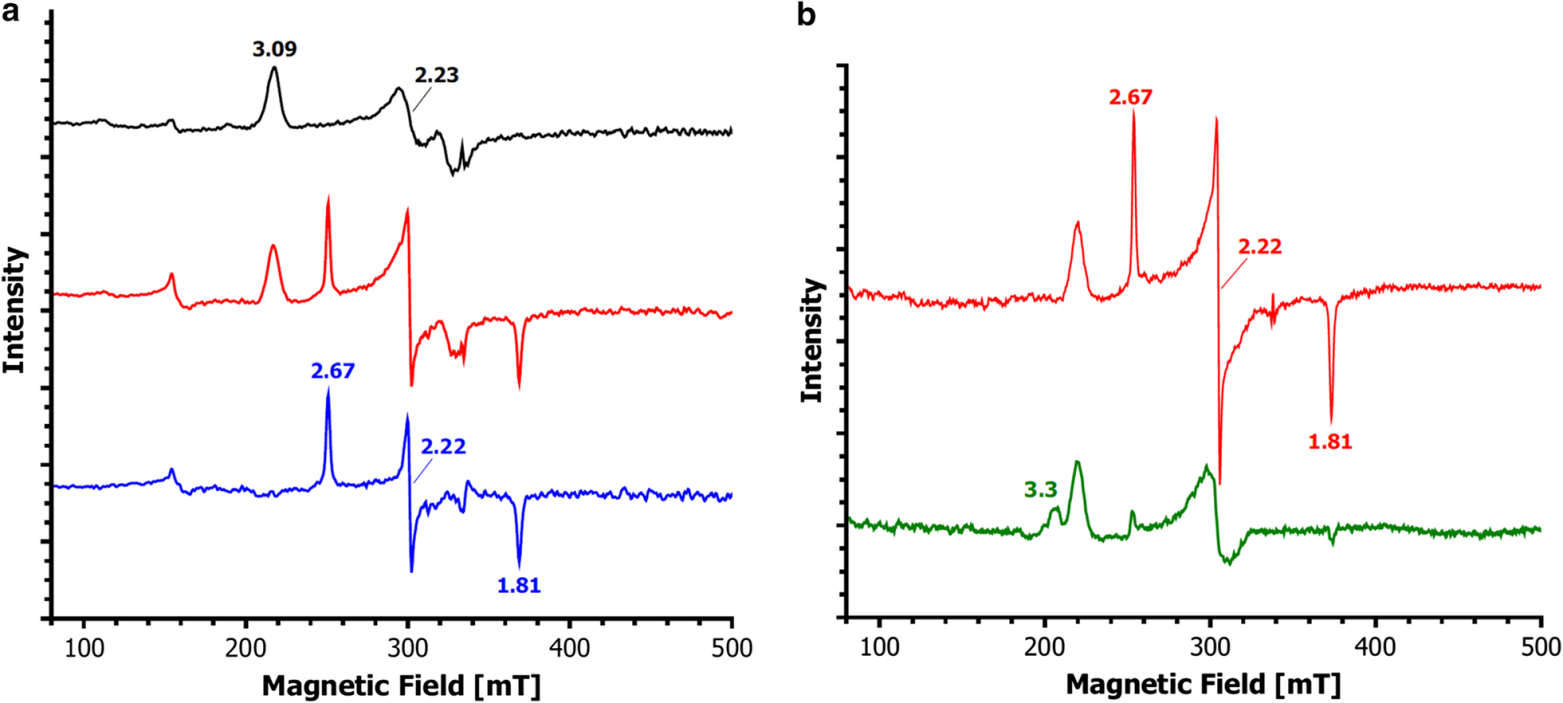
EPR-spectra of RoxA-F317Y *as isolated* compared to RoxA-Wt. **a** The difference spectrum (blue) of F317Y (red) minus the RoxA-Wt-spectrum (black) displays additional EPR-intensity representing a characteristic haem–O–Tyr coordinated *low*-*spin* species. **b** EPR-spectra of RoxA-F317Y *as isolated* (red, different preparation as in **a**) and after incubation with pyridine (5 mM, 15 min at 4 °C) (green) resulting in almost total replacement of the Tyr-ligation by pyridine

For further investigations of the haem centres of RoxA, EPR experiments were conducted. It was shown that the C-terminal haem centre of RoxA *as isolated* is present in a bis-His coordinated ferric state. Accordingly, this causes a typical rhombic EPR signal which did not change under any conditions investigated (*g *= 3.09, 2.23, ~ 1.5; (Schmitt et al. 2010; Seidel et al. 2013, Additional file 1: Fig. S3). In contrast, the signals corresponding to the N-terminal haem (active site) are predominantly absent because a major part of the active site haem is present in an EPR-silent, Fe^2+^–O_2_↔Fe^3+^–O_2_^−^ form (Seidel et al. 2013).

### Deoxygenated RoxA can be reoxygenated

Reoxygenation of RoxA, that was deoxygenated by the addition of stoichiometric amounts of the reductant dithionite, can be achieved simply by incubation under an oxygen-containing gas atmosphere (Fig. 1b). A reoxygenation of deoxygenated RoxA was also possible by the (first) addition of a slight excess of reductant (≈ twofold molar excess of dithionite to RoxA *as isolated*) and subsequent incubation under air. A high molar excess of dithionite had to be avoided since excess dithionite reacts with molecular oxygen to give oxygen-radicals that further react to hydrogen peroxide leading to the destruction of the haem porphyrin. The reaction of RoxA with hydrogen peroxide is discussed in detail later.

### External haem ligands bind to an oxygenated haem centre and displace bound dioxygen

The effects of potential haem ligands such as imidazole, pyridine or related *N*-heterocyclic compounds with free *N*-electron pairs in their molecule structures on the UV–vis spectrum and catalytic activity had been studied previously (Schmitt et al. 2010, Additional file 1: Fig. S4, Table S1). The addition of these compounds resulted in unusual and unexpected optical changes (increase of 549 α-band) and in a drastic decrease of RoxA activity. The spectral changes were reminiscent of a partial reduction of only one, the N-terminal haem centre since they affected only one of the two α-bands of RoxA (549 nm) (Schmitt et al. 2010, Additional file 1: Fig. S4). The described effects could not be well explained because of the unknown presence of a dioxygen molecule at the catalytic N-terminal haem centre at the time of the publication (in 2010). Now, the spectroscopic changes point to a displacement of haem-bound dioxygen by *N*-heterocycles that then serve as artificial ligands to a ferrous haem (schematically illustrated in the left part of Fig. 1c). This visualises the presence of a ferrous haem iron, concomitant to the reversible release a dioxygen molecule from the distal haem binding site under low oxygen pressure conditions or in the presence of low molecular weight haem ligands such as imidazole or pyridine.

Here, we investigated the effect of N-heterocyclic compounds on anaerobically reoxidised RoxA. To this end, RoxA was treated with dithionite and reoxidised by a moderate excess (< 10×) of ferricyanide under anoxic conditions (N_2_ atmosphere). This enabled us to get a RoxA preparation in a deoxygenated (bis)-ferric state. Residual chemicals were removed from RoxA by gel filtration using N_2_-saturated buffers. The effects of pyridine or imidazole (2 mM, pH 7) on this RoxA preparation were analysed by UV–vis spectroscopy (results similar for both compounds, data only shown for pyridine, Fig. 1c, right). No substantial change of the 549 nm α-band was observed even after prolonged incubation times. We conclude that pyridine (or imidazole) had bound to the oxidised (ferric), deoxygenated haem centre of RoxA. The pyridine binding to the haem group was independently confirmed by EPR spectroscopy in which binding was visualised by the occurrence of a new *low*-*spin* species with *g*_z_: 3.3 (see EPR spectrum shown later in Fig. 4b). In contrast to the reaction of anaerobically reoxidised RoxA with pyridine, the optical changes during the reaction of RoxA *as isolated* with pyridine (i.e. the intensity of pyridine-triggered “reduction bands”, exemplified by the 549 nm α-band) correlated with the intensity of the characteristic O_2_-haem α-bands at 540 and 573 nm (light blue line in Fig. 1c). The results suggest that the arising “reduction bands” after addition of N-heterocyclic compounds (imidazole, pyridine) to RoxA *as isolated* originate from a formerly reduced, oxygenated (Fe^2+^–O_2_) haem group. This feature could not be explained in the past (Schmitt et al. 2010) but can now be clarified with the presented results.

### F317 and F301 play an important role in the stabilisation of haem-coordinated dioxygen

The 3D structure of RoxA-Wt reveals exclusively hydrophobic amino acids surrounding the haem-bound dioxygen molecule (Seidel et al. 2013). In contrast to the related bacterial CCPs or MauG proteins, acid–base catalysts are absent. Instead, two phenylalanine residues (F301, F317) are located in close vicinity (4.1 and 3.7 Å) to the bound dioxygen molecule (Additional file 1: Fig. S5). To study the importance of these phenylalanines for catalytic activity, both positions were substituted by selected residues.

### RoxA-F317

Phenylalanine 317 had been previously replaced by alanine, leucine, tyrosine, tryptophan or histidine (Birke et al. 2012). Polyisoprene-cleaving activities of the F317-RoxA-variants were drastically reduced or completely abolished, and notable differences in UV–vis spectra were observed for all muteins compared to RoxA-Wt. Considering the knowledge that a dioxygen molecule is stably bound to the N-terminal haem in the RoxA molecule (Seidel et al. 2013), our findings can be now interpreted: the difference spectra of RoxA-Wt minus the RoxA-F317 muteins were recorded and revealed absorption maxima at 540 nm and 573 nm (Additional file 1: Fig. S6), very similar to chemically reduced and subsequently reoxidised RoxA (compare (Schmitt et al. 2010), Fig. 1a). These findings imply an oxidised, deoxygenated N-terminal haem centre in all characterised RoxA-F317 muteins (Fe^3+^), in contrast to the oxygenated haem of RoxA-Wt (Fe^2+^–O_2_↔Fe^3+^–O_2_^−^).

This interpretation is further supported by the reaction of RoxA-Wt and RoxA-F317-muteins with N-heterocyclic haem ligands like imidazole or pyridine (Fig. 1c, d). As discussed above, RoxA-Wt shows a substantial increase of the characteristic “reduction band” of the N-terminal haem (549 nm) upon addition of these compounds indicating a binding of pyridine or imidazole to the ferrous haem iron thereby releasing the formerly bound dioxygen molecule. In contrast, spectral changes were barely detectable in RoxA-F317Y, RoxA-F317L or RoxA-F317A upon the addition of imidazole or pyridine (Fig. 1d, Birke et al. 2012). Since the increase of the “reduction-band” at 549 nm is expected to arise from displacement of the haem-bound dioxygen as pointed out above, our findings indicate the absence of dioxygen in the active haem centre of the RoxA F317 muteins. In agreement with the interpretation of the RoxA-F317Y N-terminal haem group as being completely ferric, an exposure of RoxA-F317Y *as isolated* to a CO-saturated buffer did not change the optical spectrum, as it was also the case for anaerobically reoxidised RoxA-Wt (Additional file 1: Fig. S7). Only RoxA-Wt was able to bind CO thereby releasing the haem-bound dioxygen molecule (Additional file 1: Fig. S7). Furthermore, the RoxA-317Y variant showed some other interesting features that are described below.

### RoxA-F317Y

The optical spectrum of RoxA-F317Y displayed weak additional broad bands at approximately 610 nm and 480 nm (Fig. 1d, right image). Similar weak absorption bands have been described for a distal tyrosinate ligand in haem-containing proteins (Kraus and Wittenberg 1990; Abu Tarboush et al. 2012). In agreement with this, the absorptions around 610 nm and 480 nm were not present in the spectra of RoxA-Wt, RoxA-F317A and RoxA-F317L (Birke et al. 2012). These data suggest that the sixth ligation site of the N-terminal haem in the F317Y variant is coordinated by the tyrosine O^−^-group as tyrosinate. Moreover, the EPR spectrum of RoxA-F317Y shows signals characteristic for an oxygen-coordinated rhombic *low*-*spin* haem centre with *g*-values of *gz *= 2.67, *gy *= 2.22, *gx *= 1.81 (Fig. 2a), suggesting the presence of an oxidised tyrosinate-coordinated N-terminal haem in RoxA-F317Y. The rhombicity of this species is characteristic for a Fe^3+^–O–ligation. The *g*-values fit well with those of a tyrosinate coordination (Fe^3+^–O^−^–Tyr) at the distal binding site found at the C-terminal haem of the related MauG or other proteins (Kraus and Wittenberg 1990; Abu Tarboush et al. 2012).

To test the stability of the tyrosinate-ligation of the haem centre, we recorded an EPR spectrum after the addition of 100 µM pyridine to RoxA-F317Y. The obtained spectrum was characterised by the total loss of the rhombic Tyr-species and increase of a *low*-*spin* signal with *g*_max_ ≈ 3.3 (Fig. 2b). This spectrum resembles that of (fully oxidised) RoxA-Wt in presence of pyridine with the typical pyridine-bound haem species at *g*_max_ ≈ 3.3 (see also Fig. 4b). In conclusion, tyrosine at position 317 serves as distal ligand to the catalytic haem group confirming the results from UV–vis-spectroscopy. Furthermore, this ligation is labile since it can be displaced by pyridine.

### RoxA-F301

The experimentally demonstrated importance of residue F317 for dioxygen stabilization and catalytic activity suggests a similar function for a phenylalanine residue (F301) that is located in comparable distance to the haem iron (4.1 Å) on the opposite site of the haem-bound dioxygen (Additional file 1: Fig. S5). Two RoxA-F301 muteins (F301L and F301Y, Birke et al. 2012) were purified. The residual activities determined by the oxygen consumption assay as described previously (Röther et al. 2017b), were 21% for RoxA-F301Y and 14% for RoxA-F301L demonstrating the importance of these residues for RoxA activity. The UV–vis spectrum of RoxA-F301L is typical for an oxidised haem protein and very similar to those of the RoxA-F317 muteins. Compared to RoxA-Wt, a less pronounced absorption at 540 nm and 573 nm and a minor 549 nm band after incubation with pyridine indicates a significant but not a complete loss of haem-bound dioxygen in the *as isolated* state. Similar to RoxA-F317L, the N-terminal haem centre of the F301L mutein rests primarily in an oxidised, deoxygenated form. This emphasises the importance of the residues F301 and F317 for RoxA activity, since both are required for stabilizing the haem-bound dioxygen. However, RoxA-F301Y showed some remarkable differences compared to the other F301/F317 muteins and is described in more detail below.

### RoxA-F301Y

The polyisoprene-cleaving activity determined for RoxA-F301Y was considerably higher (21%) than it had been determined for the analogous substitution at position F317Y (< 1%, Birke et al. 2012). For further investigation, the effects of imidazole and pyridine on the UV–vis spectrum and activity of RoxA-F301Y were studied. In case of RoxA-Wt, imidazole had a similar but weaker effect as pyridine. The higher pk_a_ value of imidazole (~ 7.0) versus pyridine (5.2) leads to a higher population of protonated molecules, explaining the slower binding to the ferrous haem. Accordingly, it took longer to reach the maximum absorption at 549 nm compared to pyridine. Correspondingly, the inhibitory effect of imidazole on RoxA-Wt activity was less pronounced as it was for pyridine. Interestingly, the impact of both compounds on RoxA-F301Y was much weaker compared to RoxA-Wt (Additional file 1: Fig. S8, left).

The interaction of RoxA-F301Y with pyridine and imidazole was further studied by UV–vis spectroscopy. RoxA-Wt and RoxA-F301Y show almost identical spectra in the *as isolated* state. For RoxA-Wt, the replacement of bound dioxygen by imidazole (determined by the increase of the 549 nm band) took place within approximately 1.5 h, whereas RoxA-F301Y was not affected in this time period. After addition of pyridine, RoxA-F301Y slowly (20 min) converted to the “reduced” spectrum (increase of 549 nm absorption). For RoxA-Wt almost no additional changes were observed. The total increase of absorption at 549 nm was comparable for both proteins (Fig. 1e).

The presented data indicate a haem-bound dioxygen in RoxA-F301Y, a feature that is unique among all studied F301/F317 variants and can explain the relatively high residual activity compared to the other muteins. Moreover, the data suggest an even higher stability of the oxy-haem conformation at the N-terminal haem group compared to the wild type enzyme. This in turn is in agreement with the reduced activity compared to RoxA-Wt, since a higher stability of the dioxygen binding will decrease the substrate turnover of the enzyme. The structural reason for this additional dioxygen stabilisation probably is a consequence of the formation of a hydrogen bond of Tyr–OH with the haem-dioxygen molecule (Additional file 1: Fig. S8, right), as it is known for other haem-containing proteins (Couture et al. 1999; Yeh et al. 2000).

### Reaction of RoxA with carbon monoxide and nitrogen monoxide

Ligands commonly used to probe the coordination and oxidation state of the haem iron atom are carbon monoxide (CO), which exclusively binds to ferrous iron, and nitrogen monoxide (NO), which coordinates strongly to ferrous and with much lower affinity also to ferric iron (Cooper 1999; Helms and Kim-Shapiro 2013). The binding of CO to RoxA-Wt *as isolated* was observed by characteristic changes to the UV–vis spectrum confirming the ferrous state of haem-iron in RoxA *as isolated* (Birke et al. 2015) (Additional file 1: Fig. S7), whereas CO did not change the spectrum of ferricyanide oxidised (ferric) RoxA. The presence of CO did not change the UV–vis spectrum of RoxA (F317Y) indicating the ferric nature of the 317-O-Ligation (not shown). Since “reduction”-type signals were absent in the RoxA-Wt spectrum, the observation of a characteristic ferrous CO-adduct in RoxA-Wt can only be explained by the replacement of a dioxygen molecule by carbon monoxide leaving a ferrous haem group with bound CO (Fe^2+^–CO) whereas the optical spectrum of the oxygen-adduct in the *as isolated* state rather resembles an oxidised ferric Fe^2+^–O_2_↔Fe^3+^–O_2_ spectrum (see Fig. 1a).

**Fig. 3:**
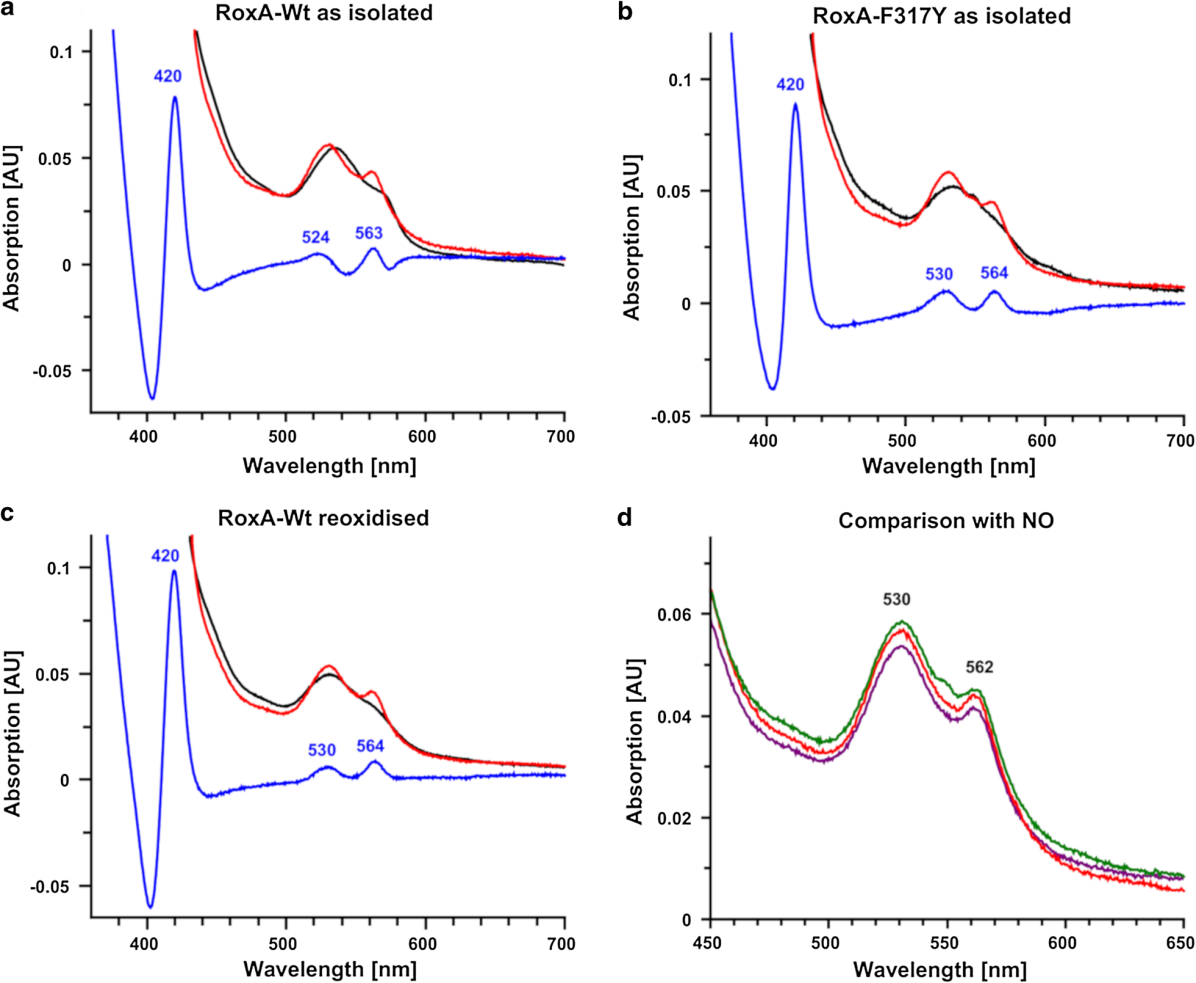
UV–vis spectra of RoxA variants in the presence of NO. The original spectra (black) of RoxAWt (**a**), RoxA F317Y (**b**) and (ferricyanide-)reoxidised RoxA-Wt (**c**) are compared with the spectra in NO-saturated buffer (red); the difference spectra (RoxA + NO minus RoxA) are shown in blue. The (fully oxidised) preparation in **b**, **c** show basically identical difference maxima while RoxA-Wt *as isolated* results in slightly distinct difference maxima caused by the presence of the oxygenated haem. **d** A direct comparison of the UV–vis Q-band region of all three preparations in presence of NO visualises their identity, however, suggesting that also the ferrous–O_2_-haem of RoxA *as isolated* (red) reacts to the same ferric NO-species as in oxidised RoxA-Wt (purple) and RoxA-F317 (green)

As expected, exposure of RoxA-Wt to nitrogen monoxide gas changed the optical spectrum resulting in new signals at 420, 530 and 562 nm, which resemble spectra of NO adducts of other haem proteins (e.g. Moir 1999; Herold and Rehmann 2003; Turner et al. 2005; Preimesberger et al. 2017). In analogy, we expected similar but distinguishable signals for Fe^2+^–NO and Fe^3+^–NO adducts. However, identical spectra to RoxA-Wt *as isolated* were observed with fully oxidised RoxA and with RoxA-F317Y in the presence of NO (Fig. 3). This leads to the conclusion that the presence of NO has led to a rupture of the Fe–O–Tyr ligation which can´t be disrupted by CO because of the ferric oxidation state of the active site haem in case of the F317Y variant. Moreover, the absence of the “reduction-bands” in optical spectra after addition of pyridine to any NO-treated RoxA-Wt sample, suggested that NO was bound to a ferric haem, contrary to (partially oxygenated) RoxA *as isolated* (see above).

We conclude that, in consequence of the reaction with NO, the oxygenated RoxA-Wt *as isolated* turns to a ferric haem which binds or further reacts with NO, rather than that an autoreduction of oxidised RoxA or F317Y would appear from a ferric nitrosyl to the corresponding ferrous adduct as it is documented for other haem proteins at higher pH (Hoshino et al. 1996). More likely, because of the ability of the respective N-terminal haem to stably coordinate dioxygen, a similar reaction as in oxygen-binding proteins may apply. Haemoglobins and other haem proteins in the oxygenated state do not form ferrous nitrosyl-haem complexes (Doyle and Hoekstra 1981) but react with NO via nitroperoxide (peroxynitrite, *low*-*spin*) as intermediate resulting in the generation of a ferric iron (*high*-*spin*) and nitrate (Eq. 1) (Gow et al. 1999) which represents an ancient function as a nitric oxide dioxygenase (Herold 1999; Su and Groves 2009, 2010; Koebke et al. 2013).

1 $${\text{Fe}}^{ 2+ } - {\text{O}}_{ 2} \leftrightarrow {\text{Fe}}^{ 3+ } {\text{O}}_{2}^{ - } + {\text{ NO}} \to \left[ {{\text{Fe}}^{ 3+ } - {\text{OONO}}^{ - } } \right] \to \left[ {{\text{Fe}}^{\text{IV}} = {\text{O}} \cdot {\text{NO}}_{ 2} } \right] \to {\text{Fe}}^{ 3+ } + {\text{NO}} _{3}^{ - } .$$

This reaction may occur in RoxA in the presence of NO and is further supported by EPR analysis. In contrast to an Fe^2+^–CO or Fe^2+^–O_2_ coordination, the Fe^2+^–NO state is paramagnetic (ferrous nitrosonium (Fe^3+^–NO/Fe^2+^–NO^+^) character) (Helms and Kim-Shapiro 2013) and therefore can be studied by EPR, whereas the ferric-nitrosyl state is EPR-silent (Sharma et al. 2017). A characteristic ferrous-nitrosyl species with typical radical signals around *g *= 2.06 (e.g. Goetz et al. 2010) was not observed by EPR of RoxA in NO-saturated buffer at 10 K. But instead of the EPR-silent NO-adducts expected from the optical spectra, an additional *low*-*spin* species with a rhombic splitting of *g*_z_ = 2.75, *g*_y_ = 2.45, and *g*_x_ = 1.53 appeared from the formerly EPR-silent dioxygen-ligated haem in RoxA *as isolated* in the presence of NO (Additional file 1: Fig. S9). The spectral changes upon the addition of NO to RoxA-Wt were appearing also for RoxA-F317Y in the same manner as the signals indicating the Tyr-ligation were decreasing (not shown). The NO-coordination disappeared both in RoxA-Wt and in RoxA-F317Y by the addition of aromatic haem ligands such as pyridine, visible at the characteristic ferric *low*-*spin* EPR-signal of pyridine-coordinated N-terminal haem in RoxA (*g*_max_ = 3.3) (Additional file 1: Fig. S9). However, for the *low*-*spin* species at *g*_z_ = 2.75, *g*_y_ = 2.41, and *g*_x_ = 1.53, both a ferrous haem-NO ligation and an EPR-silent ferric haem-NO ligation can be excluded. Possible explanations for this rhombic *low*-*spin* species seen by our EPR-assay conditions in NO-treated RoxA are discussed in the legend to Additional file 1: Fig S9. Unfortunately, such a species has not been documented for globins and remains to be elucidated.

### The reaction of RoxA with hydrogen peroxide

The stable binding of dioxygen to the active site haem of RoxA suggests a relationship of RoxAs to globins such as myoglobin and haemoglobin. Haemoglobin has a catalase-like, hydrogen peroxide consuming activity to prevent oxidative damage of the haem centre (Fielding and Langley 1958; Inada et al. 1961). All attempts to find such catalase-like activity for RoxA were not successful (Schmitt et al. 2010). Contrary, the addition of hydrogen peroxide to RoxA even at low concentrations (< 100 µM) resulted in a severe inhibition of the rubber-cleaving activity. This is somehow surprising as the core protein of RoxA has substantial structural similarity to bacterial CCPs including similar spatial orientations of both haem groups.

Recent results led to a better understanding of some previous observations of the reaction of RoxA with H_2_O_2_. The concentration-dependent inhibitory effect of H_2_O_2_ on RoxA activity presumably is due to an oxidative destruction of the catalytic (N-terminal) haem group by direct interaction of H_2_O_2_ with haem (Rinker et al. 1960), as evident from the decreasing UV–vis signals attributed to the N-terminal haem spectrum (Villegas et al. 2000). The spectroscopic changes caused by H_2_O_2_ reflect a total loss of absorbance of the oxygenated N-terminal haem of RoxA (Fig. 1f). This leads to a completely oxidised spectrum of lower intensity, which is composed by the spectrum of the oxidised C-terminal haem being unaffected and a (minor) part of the N-terminal haem which may not be accessible to external ligands (see also chapter “An unknown distal amino acid ligand can bind to the N-terminal haem centre”). The destructive effect of H_2_O_2_ is less pronounced when external ligands like pyridine or imidazole are present. Due to the rather stable ligation of N-heterocycle molecules to the N-terminal haem centre, the access for H_2_O_2_ to the distal binding site is blocked, explaining the “protective effect” of imidazole or pyridine against the destruction of the active site haem of RoxA by hydrogen peroxide. This finding suggests that the observed destructive reactions of H_2_O_2_ on RoxA are initiated by direct interaction of H_2_O_2_ with the accessible (N-terminal) haem group.

**Fig. 4:**
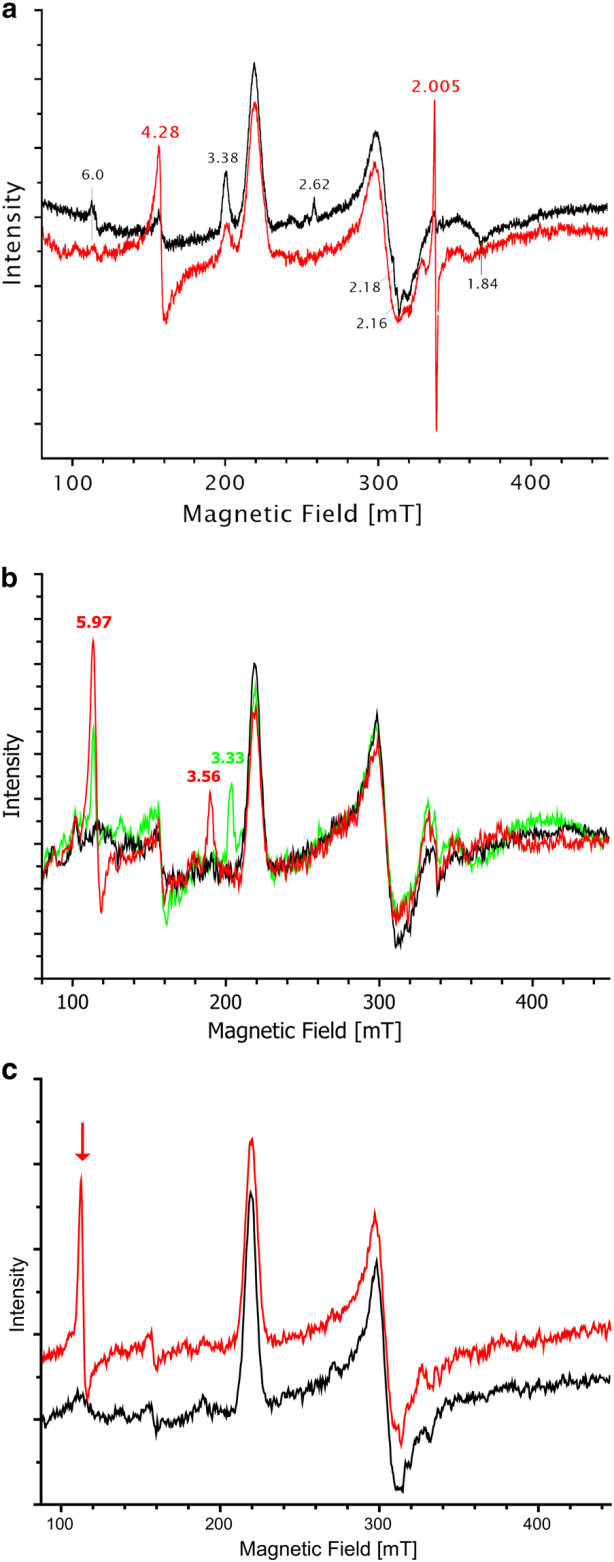
EPR spectra of RoxA. **a** Effect of hydrogen peroxide on the EPR spectrum of RoxA. EPR spectra were recorded for RoxA *as isolated* (black) and in the presence of hydrogen peroxide (red). The addition of 10 mM H_2_O_2_ leads to a rapid increase of a signal at *g *= 4.28 that indicates unbound Fe^3+^. Since the overall intensity of the EPR signal is not diminished, the unbound Fe^3+^ must have derived from a formerly EPR silent Fe^2+^ species. This illustrates that H_2_O_2_ reacts exclusively with the N-terminal Fe^2+^ haem, thereby destroying this haem centre. A minor rhombic haem species at *g *= 2.62, 2.18/2.16 and 1.84 is characteristic for a Fe^3+^–OH ligation (see below, chapter “RoxA retains its activity in the fully oxidised *low*-*spin* state”). **b** EPR spectra of RoxA *as isolated* (black), reoxidised RoxA (red, dithionite-reduced, then reoxygenated under air) and reoxidised RoxA after incubation with 5 mM pyridine (green). Reoxidised RoxA shows additional intense *low*- and *high*-*spin* ferric EPR-signals from the N-terminal haem group. The *high*-*spin* signal originates from an oxidised, fivefold coordinated haem iron (*g *= 5.97). The new *low*-*spin* signal (*g *= 3.56) is most likely caused by a nearby amino acid residue that takes the position of the previously bound oxygen molecule, establishing a sixfold coordinated haem iron. The addition of pyridine changes the *low*-*spin* signals that originate from the amino acid-ligated N-terminal haem-iron, resulting in a new ferric species with a *g*_z_-value of 3.3. **c** EPR spectra of RoxA in the absence and presence of polyisoprene. RoxA *as isolated* was incubated for 2 days in buffer (black) or in buffer supplemented with polyisoprene latex for 2 days (red) under anaerobic conditions (N_2_). The experiments indicate that a *high*-*spin* signal for ferric iron emerges from the EPR-silent ferrous-oxy state in presence of rubber latex under exclusion of dioxygen (red arrow)

The EPR spectrum of RoxA in the presence of H_2_O_2_ demonstrated that the addition of H_2_O_2_ led to an increase of a S = 3/2 signal at *g *= 4.28. Signals in this region are indicative for the presence of non-haem Fe^3+^, supporting the results from UV–vis spectroscopy and indicating a destruction of the corresponding haem centre and the release of the iron ion (Fig. 4a). Moreover, since the intensities of the other EPR signals (corresponding to the bis-His coordinated C-terminal haem centre) were not decreased, it is very likely that the signal at *g *= 4.28 arose from a formerly EPR-silent Fe species. This illustrates that H_2_O_2_ reacts exclusively with the N-terminal haem which is dioxygen-ligated (Fe^2+^–O_2_↔Fe^3+^–O_2_^−^) and EPR-silent. The (non-resolved) signal at *g*_max_ = 2.005 is a characteristic radical signal, possibly H_2_O_2_-derived or by released (non-haem) iron(III) as described by the Fenton reaction (Salgado et al. 2013) (and references cited therein).

**Fig. 5:**
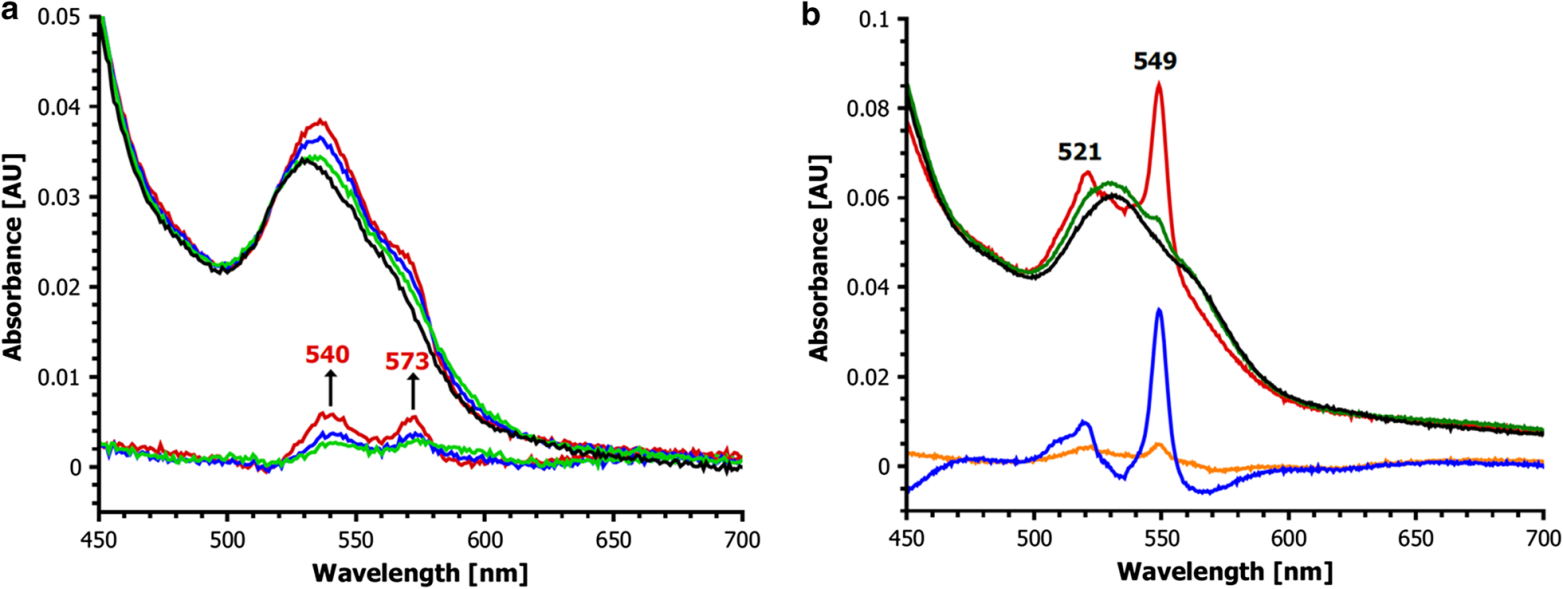
UV–vis spectroscopical changes of deoxygenated (oxidized) RoxA by hydrogen peroxide. **a** UV–vis spectra of reoxidised (deoxygenated) RoxA-Wt (black) after incubation with a tenfold stoichiometric amount of hydrogen peroxide under aerobic conditions within 15 min (black to red) and the respective difference spectra minus reoxidised RoxA. Formerly oxidised RoxA is reoxygenated in consequence of an H_2_O_2_-dependent reduction of the N-terminal haem, visible as an increase of the absorption at 540 nm and 573 nm in the difference spectrum. **b** UV–vis spectra of reoxidised RoxA-Wt (black) incubated with 0.1 mM pyridine under aerobic conditions for 1 h (green). Subsequently, stoichiometric amounts of H_2_O_2_ were added (red). Difference spectra of pyridine incubation minus reoxidised RoxA (orange) and pyridine + H_2_O_2_ incubation minus reoxidised RoxA (blue) indicate a pyridine-coordinated reduced haem in the sample of oxidised RoxA with H_2_O_2_ to the same extent as for RoxA-Wt *as isolated* (compare Fig. 1d)

Interestingly, oxidised (deoxygenated) RoxA can be reoxygenated by the presence of a slight molar excess of H_2_O_2_ within minutes. Fully oxidised (deoxygenated) RoxA was prepared by ferricyanide-treatment after dithionite-reduction under anoxic conditions and subsequent removal of excessive oxidant by gel filtration. The addition of a < 20-fold molar excess of H_2_O_2_ resulted in UV–vis bands at 540 at 573 nm characteristic for a dioxygen-ligated haem as it is found in RoxA *as isolated* (Fig. 5a). The reoxygenation was strictly depended on the presence of dioxygen excluding that H_2_O_2_ could be the source of dioxygen. Because ferrous iron is necessary to bind dioxygen, an Fe^2+^ intermediate must be involved in the reaction of the oxidised N-terminal haem of RoxA with H_2_O_2_. However, under exclusion of dioxygen no bands that indicate a reduction were observed, suggesting an immediate oxidation presumably by a direct reaction of haem with additional H_2_O_2_. To find experimental evidence for the predicted ferrous haem intermediate, the reaction of oxidised RoxA with H_2_O_2_ was examined in presence of pyridine (Fig. 5b) because of its high affinity to ferrous haem (at pH7) and our finding that this conformation was stable towards destruction by H_2_O_2_. Interestingly, this experimental approach did not result in the formation of an oxy-haem spectrum but gave evidence for the formation of a ferrous haem intermediate, as predicted. A signal typical for a reduction of haem (increase of “reduction-band” at 549 nm attributed to the N-terminal haem) appeared (for comparison see Fig. 1c, d). Oxidised RoxA was not able to form “reduction bands” in presence of pyridine when H_2_O_2_ was absent. Our observations suggest a regeneration of the oxygenated form of RoxA from the bis-ferric state in consequence of the reaction of H_2_O_2_ with Fe^3+^ leading to an Fe^2+^ intermediate state and subsequent binding of dioxygen (compare the equivalent effect of stoichiometric chemical reduction in Fig. 1b). H_2_O_2_ apparently serves as a one-electron reductant to oxidised RoxA resulting in an Fe^2+^ intermediate, which can be trapped by a ligand with high affinity to Fe^2+^ like pyridine (Fig. 5). Accordingly, in the absence of pyridine, the emergence of the oxy-haem in presence of dioxygen and low amounts of H_2_O_2_ is possible due to the high stability of this Fe^2+^–O_2_ conformation at the N-terminal haem pocket of RoxA. The mechanism for the reaction of RoxA with low amounts of H_2_O_2_ can be described by the following reactions (Eqs. 2, 3):

2 $${\text{haem}} - {\text{Fe}}^{ 3+ } + {\text{H}}_{ 2} {\text{O}}_{ 2} \to {\text{haem}} - {\text{Fe}}^{ 2+ } + {\text{HOO}} \cdot + {\text{ H}}^{ + }$$

3 $${\text{haem}} - {\text{Fe}}^{ 2+ } + {\text{O}}_{ 2} \to {\text{ haem}} - {\text{Fe}}^{ 2+ } - {\text{O}}_{ 2} \leftrightarrow {\text{haem}} - {\text{Fe}}^{ 3+ } - {\text{O}}_{2}^{ - }$$

A ferryl species, which is formed in haemoglobin with excess of H_2_O_2_ (Herold and Rehmann 2003; Keszler et al. 2008) as intermediate of a catalase-like reaction cycle (Eq. 4), was not seen in RoxA.

4 $${\text{haem}} - {\text{Fe}}^{ 3+ } + {\text{H}}_{ 2} {\text{O}}_{ 2} \to {\text{haem}} - {\text{Fe}}^{ 4+ } = {\text{O }}\left( {\text{in haemoglobin}} \right).$$

This catalase-like reaction (Eq. 4) seems unlikely to occur in RoxA, because (i) exclusively hydrophobic amino acid residues are present in the haem pocket and contradict the processing of H_2_O_2_ or the formation of a compound I equivalent as necessary for the catalase-like reaction in haemoglobin or CCPs, (ii) a destruction of haem is detectable already with low amounts of H_2_O_2_ (> 20 × RoxA) disagreeing a peroxide detoxification, (iii) the detection of an Fe^2+^ intermediate from the reaction of H_2_O_2_ with haem-Fe^3+^, which rather leads to haem destruction as from Fe^3+^ by further reaction with excessive hydrogen peroxide (Nagababu and Rifkind 2004).

Interestingly, a reduction of haem-Fe^3+^ by lipid peroxides or H_2_O_2_ has been described in literature (Peterson et al. 1981; Nagababu and Rifkind 2004). An oxygenation of oxidised haem enzymes by hydrogen peroxide has also been shown for tryptophan-2,3-dioxygenase (TDO) (Fu et al. 2011) and discussed in (Efimov et al. 2011). Since TDO oxygenation depends on the presence of its substrate L-Trp, this reaction mechanism has to differ from the reduction of RoxA by hydrogen peroxide.

### An unknown distal amino acid ligand can bind to the N-terminal haem centre

A minor, yet detectable, absorption band at 549 nm can often be seen in the UV–vis spectra of all well characterised RoxAs in the *as isolated* form (RoxA_Xsp_, RoxA_Cco_, RoxA_Mfu_, RoxA_Rgu_) (Birke et al. 2013, 2018; Jendrossek and Birke 2018), suggesting that a small fraction within a preparation is resting in a state in which the position of the haem-bound dioxygen molecules is replaced and occupied by an amino acid (compare black graph in Fig. 1a). Further evidence for an apparently (reversible) sixfold coordination of the N-terminal haem comes from a monitored chemical oxidation. As shown above, oxidation of reduced RoxA by ferricyanide leads to the loss of the oxygenation-specific bands at 540 and 573 nm in RoxA *as isolated* as a result of the conversion from the oxygenated ferrous (Fe^2+^–O_2_) to the deoxygenated ferric state (see Fig. 1a). Immediately after reoxidation of RoxA in the absence of dioxygen a weak (broad) signal at approx. 630 nm appeared (Additional file 1: Fig. S10) indicating a putative *high*-*spin* species. The signal decreased slowly within hours at room temperature (not shown). The presence of a *high*-*spin* signal indicates that the N-terminal haem is fivefold coordinated or is occupied by a weak ligand such as a water molecule at the distal binding site directly after reoxidation of RoxA. The subsequent decrease of the signal can be explained by a replacement of the water molecule by an unknown distal amino acid ligand during a prolonged incubation of the oxidised enzyme (not shown).

Independent evidence to support the assumption of a distal amino acid ligand came from EPR analysis: EPR spectra of reduced and subsequently ferricyanide-reoxidised RoxA revealed the appearance of a novel *high*-*spin* species (*g*_max_ = 5.97) as well as a novel *low*-*spin* species (*g*_max_ = 3.57) from a formerly silent ferrous conformation (Additional file 1: Fig. S3). These signals were also detected by obtaining reoxidised RoxA via reduction with an excess of dithionite and subsequent exposure to air (Fig. 4b), contrary to a reduction by stoichiometric amounts of dithionite. Reactive oxygen species (O_2_^−^, HO·, HOO·) are formed when excessive dithionite reacts with molecular oxygen, which oxidise the reduced N-terminal haem centre (see above for details) and the formerly EPR-silent species becomes visible. The new *low*-*spin* signal is most likely caused by a nearby amino acid residue that takes the position of the previously bound dioxygen molecule, establishing a sixfold coordinated haem iron (Fig. 4b, red). Furthermore, this N-terminal haem conformation was not accessible to react with small molecules such as hydrogen peroxide (not shown). However, the ligand could be displaced by strong ligands like pyridine (Fig. 4b, green) or imidazole. In EPR, the highly rhombic *low*-*spin* (HALS) signals that originate from the oxidised, amino acid-ligated N-terminal haem-iron (*g*_z_ = 3.56) change to a new ferric species with *g*_z_-values of 3.3 respectively. In contrast to hydrogen peroxide, pyridine and imidazole can remove the existing distal amino acid-ligand and serve as artificial ligands, leading to new EPR-detectable signals.

Since a lag-phase of rubber cleavage was never observed when oxidised but deoxygenated RoxA was used in the oxygen consumption assay (see chapter “RoxA retains activity in the fully oxidised *low*-*spin* state”, below), the sixfold coordination of the active site haem group must be reversed when the substrate is added in order to open the distal binding position for dioxygen and polyisoprene coordination and catalysis. In conclusion, the unusual spectral changes investigated via UV–vis- and EPR-spectroscopy confirm the presence of a stably dioxygenated state at the N-terminal haem centre of RoxA. Furthermore, our data indicate the presence of a yet unidentified amino acid ligand that can reversibly bind to the distal position of the N-terminal haem group, thereby preventing the haem to interact with reactive molecules such as hydrogen peroxide. N-heterocyclic ligands like pyridine and imidazole can displace the amino acid ligand under the investigated conditions (Figs. 4b, 5b), suggesting a high flexibility at the distal haem pocket, where substrate cleavage is supposed to occur. As a consequence, we propose a structural change in RoxA triggered by substrate binding or haem reduction in the presence of substrates.

### RoxA retains its activity in the fully oxidised *low*-*spin* state

The experiments presented above confirm the distal binding pocket of the N-terminal haem as the catalytic centre where the oxidative cleavage of poly(*cis*-1,4-isoprene) occurs. The spectroscopic studies of the different oxidation states of RoxA and its interaction with various substrates and ligands described above provide a better understanding of the chemical properties of the catalytic haem group and enable us to re-evaluate the postulated mechanism of poly(*cis*-1,4-isoprene) cleavage, in which a reduced, oxygenated haem centre is needed for catalytic activity. In the previously postulated reaction mechanism (Seidel et al. 2013; Ilcu et al. 2017), an oxidised (and thus deoxygenated) haem centre would lack the ability to cleave polyisoprene. Nevertheless, when we compared the activities of RoxA *as isolated* and oxidised RoxA via the HPLC- or the oxygen-consumption assay, no significant differences were found. Obviously, oxidised RoxA retains its activity in the presence of the natural substrate by a yet unknown mechanism.

EPR experiments indicated that ferric haem emerged from the ferrous-oxygenated state in the presence of polyisoprene latex under exclusion of dioxygen. Additional EPR intensity was seen exclusively within a *high*-*spin* species (Fig. 4c). This formation of new *high*-*spin* signal intensity was not observed in the absence of latex and barely when dioxygen was present at the same time with rubber latex. These observations suggest that ferric haem iron could appear as intermediate of rubber cleavage or, at least, RoxA undergoes an oxidation in the absence of dioxygen, possibly to prevent damage to the active site haem. For the same reason, the (reversible) ligation of an amino-acid ligand may be favoured in the absence of the substrate poly(*cis*-1,4-isoprene). Presumably, an initial reduction of the oxidised RoxA is needed for (re)activation, since only reduced haem is able to bind a dioxygen molecule. For instance, RoxA could abstract an electron from the substrate, but we could not find evidence that indicates an electron transfer from polyisoprene latex to oxidised RoxA, putatively leading to the disappearance of EPR-signals from the N-terminal haem upon reduction or the appearance of a substrate radical. However, a substrate radical may be short-lived and thus would be not visible under the conditions applied in EPR spectroscopy. A signal with *g *= 2.16, previously detected when RoxA was incubated with substrate analogues (β-carotene, α-tocopherol, squalene, pristane) (see Fig. 4c of Schmitt et al. 2010), was suggested to represent an enzyme-bound substrate radical. However, our further investigation revealed this signal to be the *g*_y_ component of a rhombic *low*-*spin* signal originating from the catalytic N-terminal haem, which is supposed to be in an Fe^3+^–O–X ligation with *g*-values of 2.62, 2.16 and 1.86, typical for an OH^—^coordinated state (Additional file 1: Fig. S11) and very similar to the Fe^3+^–O–Tyr ligation present in RoxA-F317Y (compare Fig. 2). β-Carotene and α-tocopherol inhibit RoxA presumably because both are chemically related to polyisoprene but are not cleaved by RoxA. Since the native substrate, polyisoprene, is insoluble in aqueous solution, its cleavage by RoxA can hardly be studied by UV–vis spectroscopy. Nevertheless, the observed changes of EPR spectra of RoxA in the presence of polyisoprene and inhibitory substrate analogues indicate that the substrates bind to RoxA in direct vicinity of the N-terminal haem group and may react with a bound dioxygen molecule in an Fe^3+^–O-substrate-analogue dead end product. By addition of such small hydrophobic substrate analogues *low*-*spin* signals of a strong-ligand-coordination (amino acid) were decreasing or altered in *g*-value (Additional file 1: Fig. S11), which confirms a change in the electronic environment around the catalytic haem centre, e.g. by a differing ligand angle to the porphyrin plane or ligand removal.

## Discussion

In this contribution we have re-evaluated spectroscopic UV–vis and EPR data of RoxA-Wt and RoxA muteins with amino acid exchanges in the active site in the light of the meanwhile available high-resolution structure of RoxA and have determined the spectroscopic properties of some newly constructed RoxA muteins. In particular, the increase of absorption in the region of the active site haem α-band at 549 nm upon the addition of low molecular ligands such as various *N*-heterocycles (e.g. imidazole, pyridine and others) and the reaction of RoxA with hydrogen peroxide, carbon monoxide and nitrogen monoxide can now be explained: RoxA which has been isolated without previous contact to its substrate poly(*cis*-1,4-isoprene), does stably coordinate a dioxygen molecule at the distal binding site of the active site haem centre. The haem-bound dioxygen molecule can be replaced by these compounds thereby fixing the Fe^2+^–O_2_↔Fe^3+^–O_2_^−^ equilibrium of the active site haem to the (reduced) ferrous state. The finding of a stable His-Fe^2+^–O_2_ haem coordination has been already supported by structural data and the observation of additional EPR-intensity raising after reoxidation from the EPR-silent oxygenated haem (Seidel et al. 2013). We provided evidence that this coordination is, in the absence of rubber substrate, remarkably stable and reversible, as it can be regenerated from fully oxidised RoxA either by chemical reduction of the haem centre in presence of dioxygen or by an H_2_O_2_-triggered one-electron transfer reaction. This is untypical for a *c*-type cytochrome, but reasonable for a dioxygenase. However, it distinguishes RoxA from other haem-dependent oxygenases. Despite the permanent binding of dioxygen to the active site in RoxA *as isolated* the protein is a remarkably stable enzyme as long as hydrogen peroxide is absent. Only the catalytic N-terminal haem centre is sensitive to destruction by an excess of H_2_O_2_. However, RoxA—in the absence of dioxygen—can protect its active site haem from oxidative destruction by the (reversible) binding of either a not yet identified internal amino acid residue or by small ligand molecules such as imidazole.

Another outcome of this study was the finding that the two in RoxA proteins conserved aromatic residues close to the active haem distal site (F301 and F317) are important for the stable binding and correct positioning of the haem-coordinating dioxygen molecule in order to cleave poly(*cis*-1,4-isoprene). The F301Y variant is supposed to stabilise dioxygen even better than RoxA-Wt, but affects the rubber cleavage negatively. Previously, we showed that in other RoxA muteins such as F317A, F317L, F317Y or F317W the dioxygen-coordination is destabilised, dependent on both hydrophobicity and size of the residue, resulting in poor or missing catalytic activity (Birke et al. 2012). The strong hydrophobic nature of the distal haem environment and the absence of any acidic residues and acid–base catalysts distinguishes RoxA from other haem-dioxygenases like tryptophan-dioxygenase (TDO) or indoleamine-dioxgenase (IDO) and also from other proteins which can stably bind dioxygen like globins. The significant differences to structurally related bacterial di-haem *c* peroxidases we have previously discussed in (Seidel et al. 2013). The oxygen-transport protein haemoglobin (*b*-type) exhibits an overall similar hydrophobic distal haem cavity, but has a His-residue in a similar position as F317, which stabilises the dioxygen-coordination by a hydrogen-bonding interaction and can act as an acid–base catalyst. Its exact position is critical for the function of haemoglobin (e.g. Momenteau and Reed 1994). In contrast, the F317H variant of RoxA is inactive (Birke et al. 2012). Accordingly, this indicates the exact position of F317 and, as we demonstrated here, also the position of F301 to be critical for the rubber cleaving activity in RoxA by stabilising the oxygenated state in a manner necessary for oxidative rubber cleavage. Indeed, Phe has been shown to fulfil this purpose also in oxymyoglobin. An exchange of Leu29 against Phe, being in similar distance to the haem iron as in RoxA, displayed a 15-fold higher affinity to dioxygen (Carver et al. 1992), stabilising the oxy complex and resulting in an unusually low autoxidation rate. The L29F variant stabilises the O_2_-coordination by favourable electrostatic interactions between the polar O_2_ ligand and the multipole of the Phe29-phenyl ring (Watanabe et al. 2018).

TDO and IDO belong to the few other well-characterised haem-*c* dioxygenases (Basran et al. 2016). For both of them, oxy-haem compounds have been also described. However, they are much less stable than  in RoxA, for example the half-live times of oxy-haem compounds in TDO (Basran et al. 2008) and in IDO (Chauhan et al. 2008) were in the range of seconds to minutes. Recently, direct evidence for the presence of a ternary Fe(II)–O_2_-tryptophan (substrate) complex was obtained spectroscopically in TDO (Basran et al. 2016). Unfortunately, spectroscopic analysis of RoxA in the presence of its (milky) substrate polyisoprene is not possible. TDO and IDO are isolated in the oxidised state and need an initial reduction to bind dioxygen. During the cycle of substrate cleavage, no further reduction is needed as in the case of peroxidases. This may also apply to RoxA, but without the need for an initial reduction of a ferric haem in order to bind dioxygen like in TDO or IDO dioxygenases. However, since RoxA retains its activity in the fully oxidised and sixfold coordinated state, we hypothesise that a (possibly optional/second) activation pathway also exists for RoxA. The source of the electron necessary for re-oxygenation and the impact on the molecular reaction mechanism and possible conformational rearrangements relating thereto, have been subject of speculation since a long time and are still pending issues. Remarkably, our experiments provide evidence that the active site environment in RoxA has evolved to strongly stabilise a dioxygen molecule in the resting state or during the reaction mechanism. Moreover, the stability of this conformation is directly correlated to the substrate cleaving activity indicating that the stable positioning of O_2_ is essential.

The exact position of the (polyisoprene) substrate binding site in RoxA is still unknown as all attempts to co-crystallise RoxA-Wt or inactive RoxA (F317A) with the C_15_-(12-oxo-4,8-dimethyl-trideca-4,8-diene-1-al, ODTD), C_20_- or related oligoisoprenoids derived from cleavage of rubber with Lcp from *Streptomyces* sp. K30 (Röther et al. 2017a) were not successful. Therefore, further biochemical and molecular biological analysis of polyisoprene cleavage by RoxA or related rubber oxygenases is necessary. Recombinant *S. rubberoxidans* 35Y strains (Table 1) for the high level expression of *roxA* are available from the corresponding author upon request.

## Supplementary information

**Additional file 1: Table S1.** Effect of potential external haem ligands like imidazole and related low molecular compounds on activity and on UVvis-properties of RoxA-Wt as isolated. **Figure S1.** RoxA incubated with ferricyanide (left) or pyrogallol (right). **Figure S2.** UVvis spectra of RoxA. **Figure S3.** EPR spectra of RoxA. **Figure S4.** RoxA incubated with different haem ligands. **Figure S5.** Part of the RoxA active site. **Figure S6.** Comparison of UVvis sprectra of RoxA Wt and RoxA-F317A. **Figure S7.** Reaction of RoxA-Wt and RoxA-F317Y with carbon monoxide. **Figure S8.** (left) Effect of pyridine and imidazole on the activity of RoxAF301Y. **Figure S9.** EPR spectra of RoxA. **Figure S10.** Optical spectrum of RoxA after reduction and reoxidation under anaerobic conditions (enlarged on the right). **Figure S11.** EPR spectra of RoxA-Wt in the presence of small substrate analogues.

## Data Availability

Not applicable.
