# Supplementary material for: Towards the understanding of the enzymatic cleavage of polyisoprene by the dihaem-dioxygenase RoxA
Source: AMB Express. 2019 Oct 17;9:166. doi: 10.1186/s13568-019-0888-0 (PMC6797691; doi:10.1186/s13568-019-0888-0)
Supplement: Supplementary file 1 — Additional file 1: Table S1. Effect of potential external haem ligands like imidazole and related low molecular compounds on activity and on UVvis-properties of RoxA-Wt as isolated. Figure S1. RoxA incubated with ferricyanide (left) or pyrogallol (right). Figure S2. UVvis spectra of RoxA. Figure S3. EPR spectra of RoxA. Figure S4. RoxA incubated with different haem ligands. Figure S5. Part of the RoxA active site. Figure S6. Comparison of UVvis sprectra of RoxA Wt and RoxA-F317A. Figure S7. Reaction of RoxA-Wt and RoxA-F317Y with carbon monoxide. Figure S8. (left) Effect of pyridine and imidazole on the activity of RoxAF301Y. Figure S9. EPR spectra of RoxA. Figure S10. Optical spectrum of RoxA after reduction and reoxidation under anaerobic conditions (enlarged on the right). Figure S11. EPR spectra of RoxA-Wt in the presence of small substrate analogues. [file 13568_2019_888_MOESM1_ESM.pdf]

AMB Express

Towards the Understanding of the Enzymatic Cleavage of Polyisoprene by the Dihaem-Dioxygenase RoxA

Additional file 1

Georg Schmitt, Jakob Birke and Dieter Jendrossek*

Institute of Microbiology, University of Stuttgart, Germany

[*]present address: Institute of Applied Biotechnology, University of Applied Sciences Biberach, Hubertus-Liebrecht-Strasse 35, 88400 Biberach, Germany

*Correspondent footnote: Dieter Jendrossek

Institut für Mikrobiologie
Universität Stuttgart
Allmandring 31
70569 Stuttgart
Germany
Tel.: +49-711-685-65483
Fax: +49-711-685-65725
E-mail: dieter.jendrossek@imb.uni-stuttgart.de
or imbdj@imb.uni-stuttgart.de

Additional file 1: Table S1: Effect of potential external haem ligands like imidazole and related low molecular compounds on activity and on UVvis-properties of RoxA-Wt *as isolated* (originally published in Schmitt et al. 2010)**.**

Compound	Structure	Maximum (Soret-band) [nm]	Maximum (Soret-band) in difference spectrum [nm]	Intensity of Soret band in difference spectrum [mAU]	Velocity of effect	Residual activity [%]			
						10mM	1mM	100µM	10µM
**Imidazole**	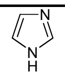	407 + 417	418	140	fast	< 10	15	45	85
**1-Methyl-imidazole**	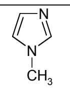	407 + 417	418	145	fast	< 10	< 10	40	nd
**2-Methyl-imidazole**	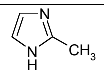	407	(418)	10	slow	75	> 95	> 95	nd
**1,2-Dimethyl-imidazole**	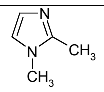	407	(418)	10	very slow	50	90	> 95	nd
**Pyrazole**	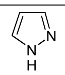	412	416	90	very fast	< 10	45	90	nd
**(3,5)- Dime-thylpyrazole**	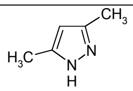	407	415-416	25	slow			95	90
**Pyrrole**	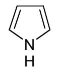	407	-	< 5	no effect		> 95	> 95	nd
**Pyrazine**	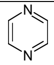	411	415	70	very fast	10	10	30	nd
**2-Methyl-pyrazine**	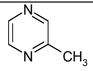	411	415	90	very fast		10	40	nd
**2,5-Dimethyl-pyrazine**	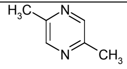	407	414	10	no effect		90	95	nd
**Pyridine**	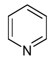	413-414 (with shoulder at 407)	416	100	very fast	< 5	5	10	nd
**2-Methyl-pyridine**	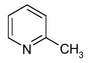	407	-	< 5	no effect		85	90	nd
**4-Methyl-Pyrimidine**	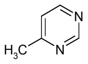	412	415	100	medium		10	35	nd
**Pyridazine**	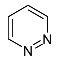	410	415	60	fast		45	90	nd
**Pyrrolidine**	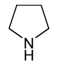	411	418	85	slow		80	90	nd
**Methimazole**	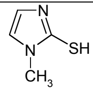	407	-	< 5	no effect	95	> 95	> 95	nd
**Hydrazine**	$H_2N - NH_2$	407 (general decrease of absorption)	416	10	slow	< 5	< 5	< 5	5

Results of UVvis spectroscopy and activity assays of RoxA in the presence of imidazole and structurally related, N-containing compounds under oxic conditions are given. RoxA as isolated has a Soret maximum at 407 nm. All optical investigations were performed at a RoxA concentration of 2 µM. The Soret maxima in the absolute UVvis spectrum and the spectral changes in difference spectrum [RoxA in the presence of the test compound minus RoxA as isolated] are listed, as well as the order of velocity of the effects. The intensity of the change of Soret band in difference spectrum can be compared to a total intensity of about 350 mAU with fully reduced RoxA (Na-dithionite) at 418 nm. Activity assays were performed as described in the methods section in 100 mM potassium phosphate buffer (pH 7.0) with incubation for 3 hours. The residual activities are shown in % of the untreated control (RoxA without compounds). Not determined (nd), no significant change (-).

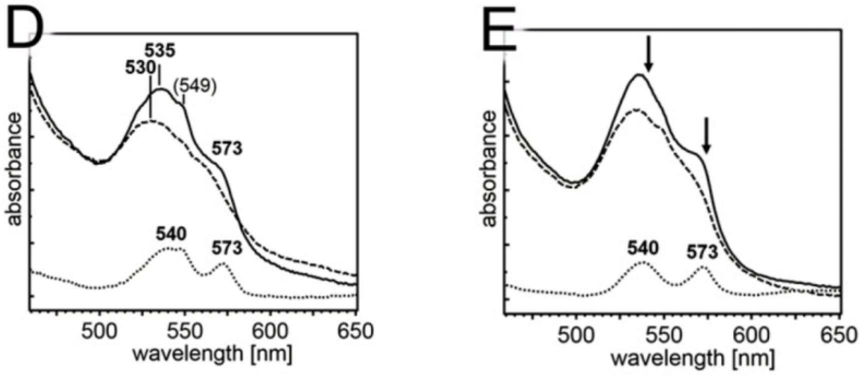

Additional file 1: Fig. S1: RoxA incubated with ferricyanide (left) or pyrogallol (right) (originally published in Fig. 4D, E of (Seidel et al. 2013))

After incubation of RoxA *as isolated* (solid line) with ferricyanide, distinct UVvis features that are typical for a Fe^{3+} spectrum are observed (dashed line). The difference spectrum (dotted line) visualises signals at 540 and 573 nm.

A similar effect is observed when RoxA *as isolated* (solid line) is treated with pyrogallol to remove bound O_2 (dashed line). Arrows indicate a loss of absorption on removal of dioxygen.

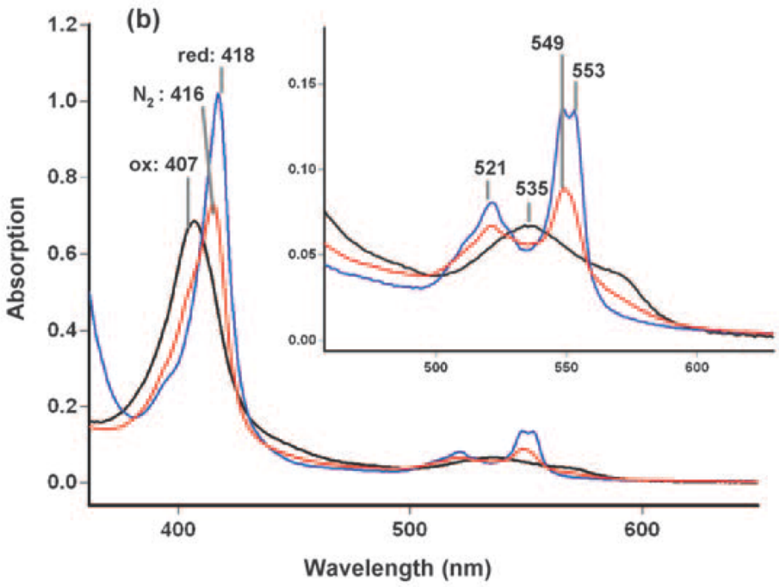

Additional file 1: Fig. S2: UVvis spectra of RoxA.
RoxA *as isolated* (black), dithionite-reduced (blue) and incubated under N_2 atmosphere (red) for 48h (originally published in Fig. 1b of (Schmitt et al. 2010)). The increasing 549 nm α-band visualises a pseudo-reduction of the N-terminal haem centre under low oxygen gas pressure because of the reversible removal of dioxygen. In this case, a ferrous iron is left that can be reoxygenated under air atmosphere.

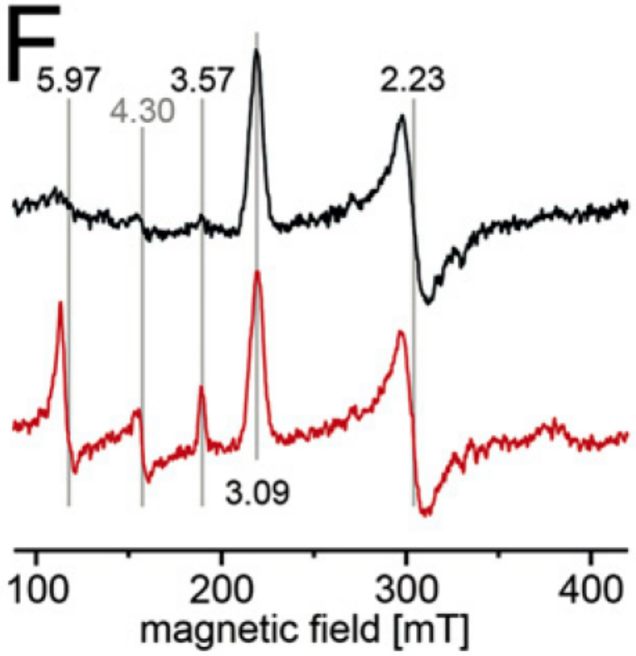

Additional file 1: Fig. S3: EPR spectra of RoxA.
RoxA *as isolated* (black) and reoxidised RoxA (red, dithionite reduced and subsequently reoxidised with ferricyanide) (originally published in Fig. 4F of {Seidel:2013eu}). The C-terminal haem group rests in the oxidised state, therefore it can be seen in the *as isolated* spectrum (g= 3.09, 2.23, ~1.5). The reduced, dioxygen bound N-terminal haem centre is EPR silent (black). After reoxidation, this haem is visible as two different species, one *high spin* state that probably corresponds to a 5-fold ligated state (*g*=5.97) and a new low spin species (*g*=3.57) that is most likely 6-fold coordinated (red). The sixth coordination sphere might be occupied by an unknown distal ligand. The signal at *g*=4.3 refers to non-specifically bound iron(III).

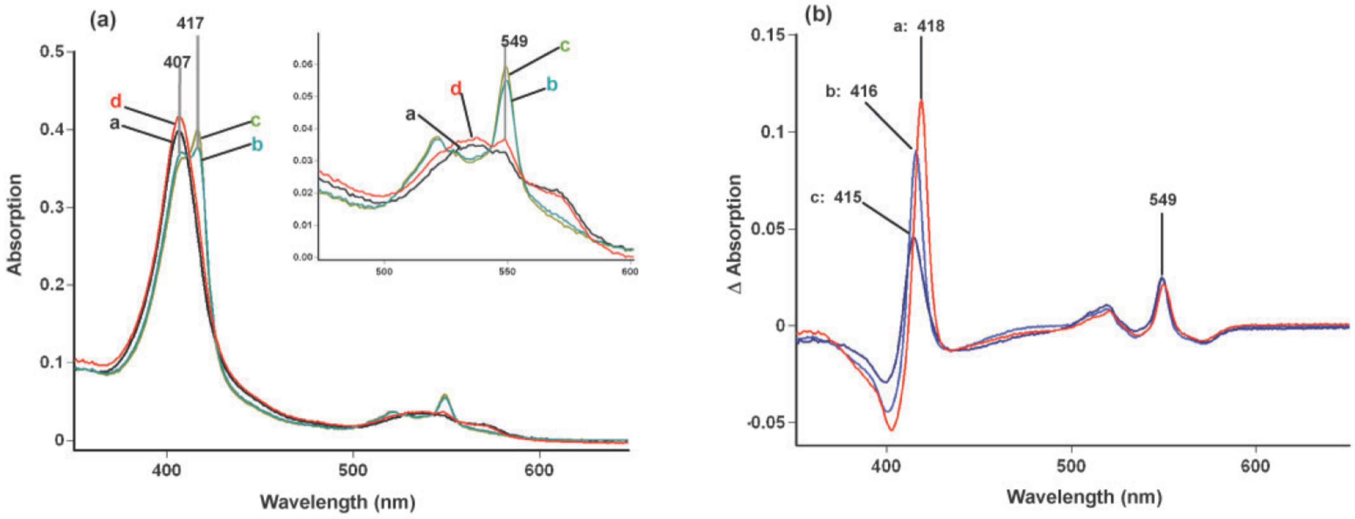

Additional file 1: Fig. S4: RoxA incubated with different haem ligands
(originally published in Fig. 3 of (Schmitt et al. 2010)).

(a) UV/vis spectra of RoxA *as isolated* (a, black line) and RoxA in the presence of 1 mM imidazole (b, blue), 1-methylimidazole (c, green) and 2-methylimidazole (d, red), incubated for 1 h at room temperature under air. A double Soret maximum at 407 and 417 nm and an α-band at 549 nm (inset) appeared after the addition of imidazole and 1-methylimidazole.

(b) Difference spectra (RoxA-ligand minus RoxA *as isolated*) with imidazole (a, red), pyridine (b, black) and pyrazine (c, blue) at the end point of the reaction (all under air atmosphere).

The effects of most ligands (with exception of 2-methylimidazole) can be explained by ligand binding to the N-terminal haem centre, thereby substituting dioxygen as axial distal ligand. As a result, the spectra show characteristics of an oxidised (C-terminal, Fe^{3+}) as well as a reduced state (N-terminal: Fe^{2+}-ligand). 2-methylimidazole cannot substitute O_2, possibly the methyl group leads to a steric hindrance that prevents a binding to the haem.

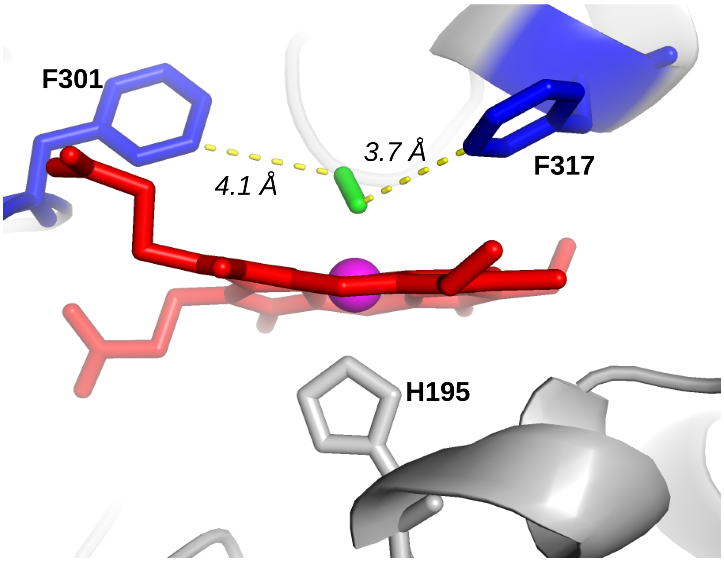

Additional file 1: Fig. S5: Part of the RoxA active site. The distal haem pocket of the catalytic N-terminal haem centre (red) consists of hydrophobic amino acid side chains. The residues F301 and F317 (blue) are in close vicinity to the haem-bound dioxygen molecule (green). The two axial ligands are shown in grey (H195) and green (dioxygen).

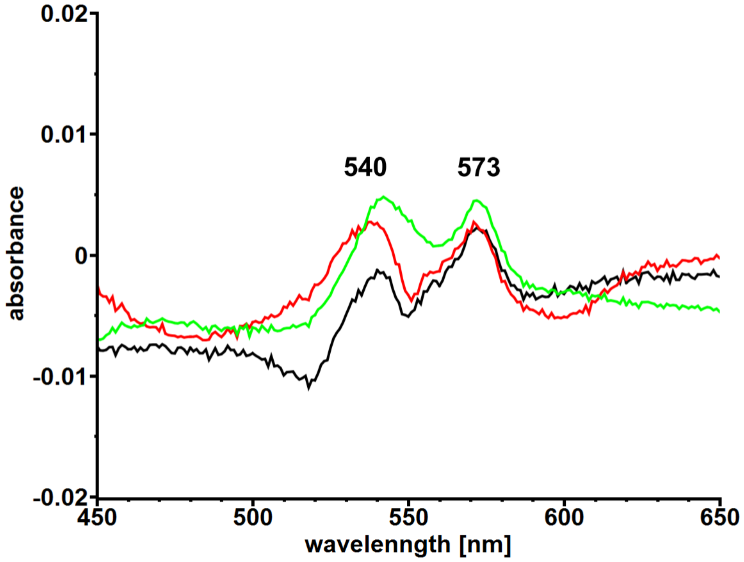

Additional file 1: Fig. S6: Comparison of UVvis sprectra of RoxA Wt and RoxA-F317A. UVvis difference spectra of RoxA-Wt minus RoxA-F317L (black), RoxA-F317Y (red), RoxA-F317A (green) in the Q-band regions. The difference spectra show decreased absorptions of the muteins at 540 nm and 573 nm. These are characteristic for oxidised N-terminal haem centres, thus without bound dioxygen as in RoxA-Wt as isolated (compare **Fig. 1**).

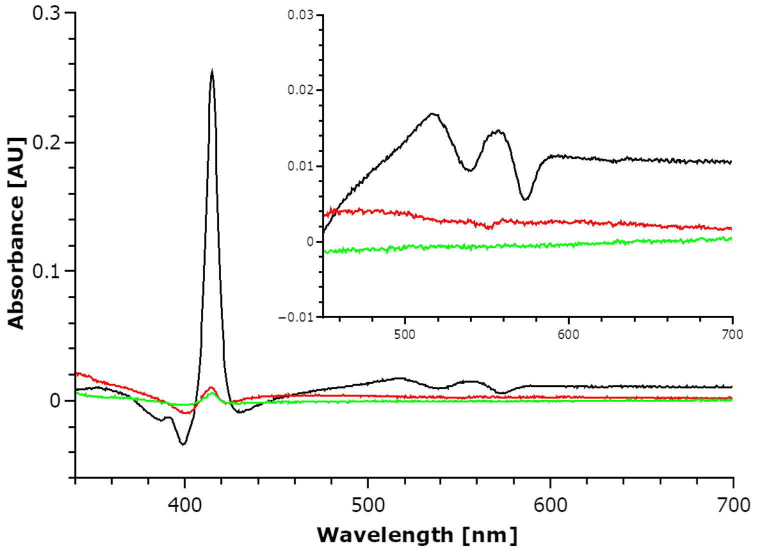

Additional file 1: Fig. S7: Reaction of RoxA-Wt and RoxA-F317Y with carbon monoxide. UVvis difference spectrum of RoxA-Wt incubated with CO-buffer minus RoxA-Wt as isolated (compare Fig. 6 of (Birke et al. 2015)), oxidised RoxA-Wt + CO minus oxidised RoxA-Wt (red) and RoxA-F317Y + CO minus RoxA-F317Y as isolated (green). The Q-Bands are enlarged (inset). The spectral changes indicate a release of haem-bound dioxygen and a binding of CO to the ferrous N-terminal haem centre of RoxA-Wt. Only minor changes appeared with both oxidised RoxA-Wt and RoxA-F317Y, which indicates the ferric nature of the respective haem centre.

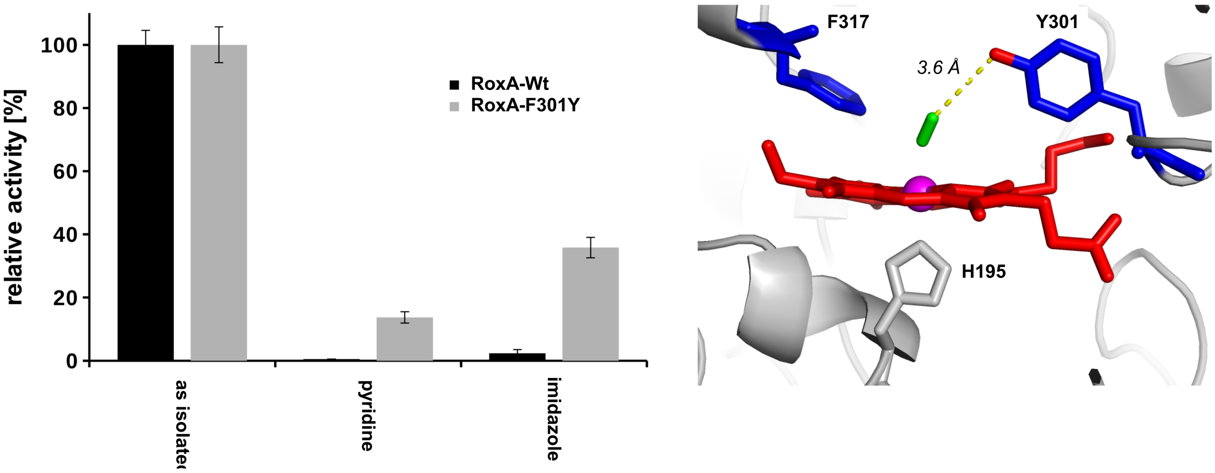

Additional file 1: Fig. S8: (left) Effect of pyridine and imidazole on the activity of RoxA-F301Y. Relative activities of RoxA-Wt and RoxA-F301Y (both set to 100%) in the presence of pyridine or imidazole (2 mM). The activity of RoxA-F301Y was 21% of RoxA-Wt. The haem ligands have a weaker effect on the activity of RoxA-F301Y compared to RoxA-Wt. **(right)** The haem pocket of the N-terminal haem centre of RoxA-F301Y (different view compared to **suppl. Fig. S6**). The structure was modelled with the SWISS model server (Biasini et al. 2014) based on the RoxA-Wt structure (pdb: 4B2N). Tyrosine 301 stabilises the haem-bound dioxygen molecule (green) with a hydrogen bond, leading to decreased activity.

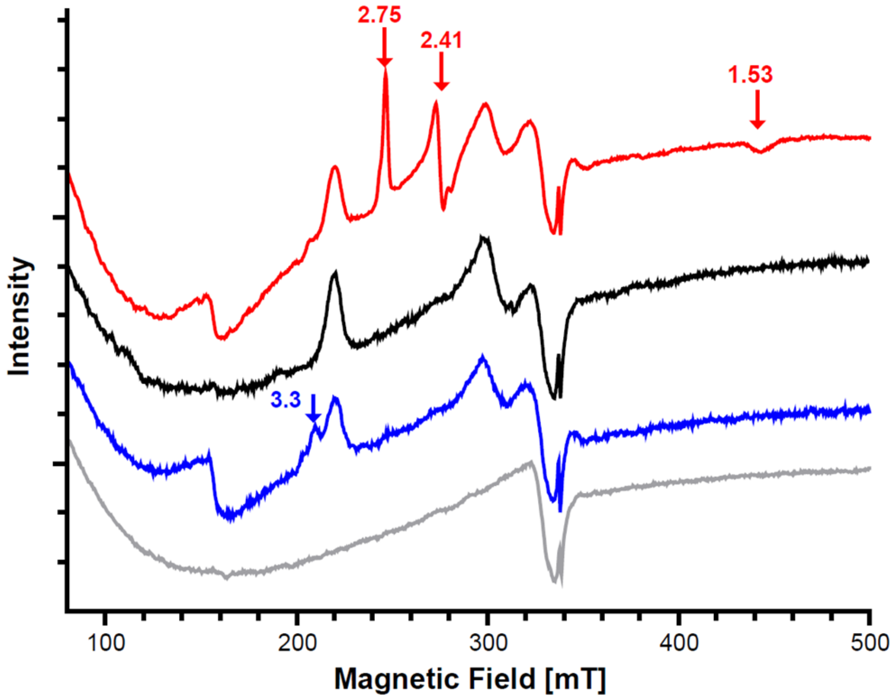

Additional file 1: Fig. **S9**: **EPR spectra of RoxA.** The spectra were recorded at 10 K of RoxA *as isolated* (black), RoxA in NO-saturated buffer (red, 5 min) and this sample after addition of pyridine (5 mM, 15 min) (blue). Reaction of RoxA as isolated with NO resulted in a new rhombic species at g_z=2.75, g_y=2.41, and g_x=1.53. This species completely disappeared by addition of pyridine while the characteristic pyridine-ligated low-spin signal at g=3.3 formed at the same time. These spectra are shown without cavity-subtraction for a better resolution of the haem signals. For comparison, a spectrum of buffer only is shown (gray).

Besides in porphyrin models in organic solvents such a rhombic low temperature species at g_z=2.75, g_y=2.45, and g_x=1.53 has not been described for a NO-derived enzyme-bound haem species, so far, to our knowledge. This species is much distinct to a ferrous-NO ligation, the anisotropy is similar to a nitrite-coordination and rather reminds of a peroxynitrite ferrihaem complex (Sharma et al. 2017). Interestingly, bis-(nitro-)*low-spin* species as described with porphyrin model systems (Munro and Scheidt, 1998; Nasri et al., 1990; Lyakin et al. 2009) fit very well the observed species, but comparability to RoxA is limited. The binding of nitric oxide often leads to lability or even rupture of the *proximal* (His-) N-Fe bond (e. g. e.g. Reynolds et al. 2000; Rodgers et al. 2000;), the generation of a bis-NO_2^--coordination at the N-terminal haem of RoxA is unlikely.

We provided evidence that a ferric N-terminal haem is formed in a first reaction from RoxA-WT (O_2-ligated) with NO. Since *high-spin* signals were completely absent in the respective EPR- and UVvis spectra, we propose a further reaction of this ferric haem with additional NO or NO_2^-/NO_3^-, which could be formed by reaction of ferric haem with NO resulting in nitrite (NO + H_2O/OH^- -> NO_2^-). Also, a reaction of the proposed intermediate peroxynitrite (eq. (1)) with Phe317 close to the distal coordination site in RoxA WT or Tyr in RoxA-F317Y must be taken into account: nitrophenylalanine or *p*-nitrotyrosine, respectively, could be formed (Beckmann and Koppenol, 1996; Alvarez and Radi, 2003) and serve as the haem distal ligand. Because the UVvis signals could be interpreted as ferric-NO derived, but not the EPR spectra, a distinct conformation appearing upon freezing to low temperature (10 K) may be considered.

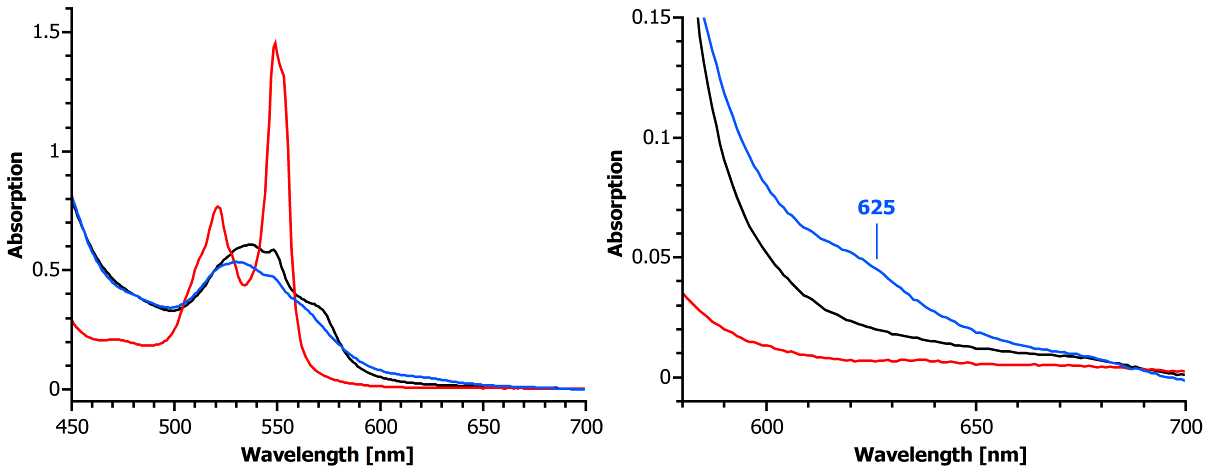

Additional file 1: Fig. S10: Optical spectrum of RoxA after reduction and reoxidation under anaerobic conditions (enlarged on the right). When RoxA *as isolated* (black) is reduced (red) and subsequently ferricyanide-reoxidised (blue), an additional weak absorption increase upon ferricyanide-reoxidation around 625 nm can be observed, indicating a *high-spin* coordination at the N-terminal haem centre directly after reoxidation.

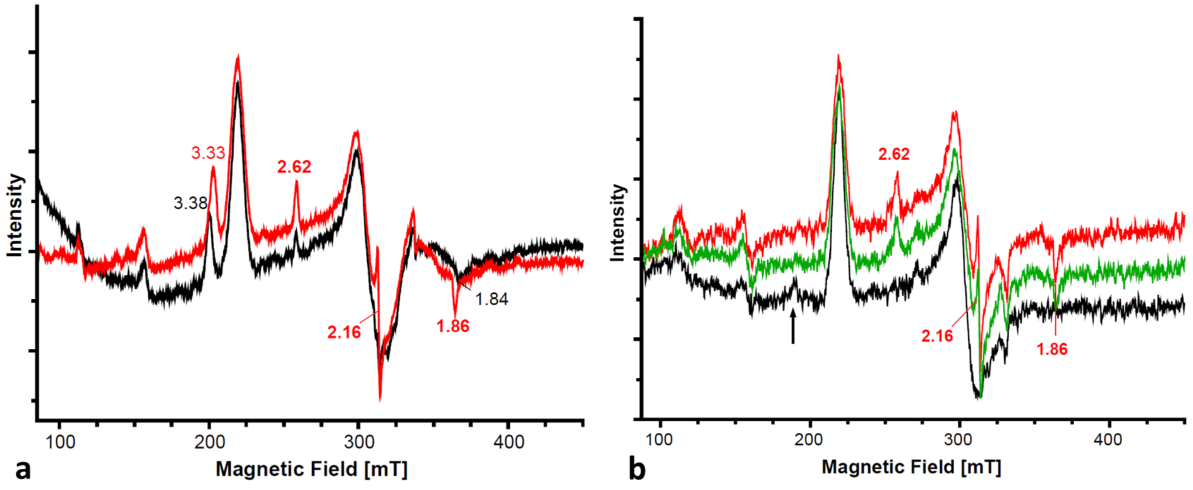

Additional file 1: Fig. S11: **EPR spectra of RoxA-Wt in the presence of small substrate analogues.** **(a)** RoxA-Wt as isolated from rubber latex culture (black) and after addition of β-carotene (2 mM, red). A rhombic *low-spin* species with *g*-values of 2.62, 2.16 and 1.86, indicating a haem-O-X coordination, was increasing in presence of β-carotene and another low-spin species at *g*=3.38 (strong ligand), attributed to the same (N-terminal) haem, was shifted. **(b)** From recombinant RoxA-Wt (black) the same species at *g*=2.62, 2.16 and 1.86 appeared by addition of pristane (2 mM, red) or squalene (2 mM, green), respectively, and a minor *low-spin* signal (black arrow) disappeared.

References

Alvarez B and Radi R. (2003) Peroxynitrite reactivity with amino acids and proteins. *Amino Acids* 25:295–311. doi: 10.1007/s00726-003-0018-8

Biasini, M., Bienert, S., Waterhouse, A., Arnold, K., Studer, G., Schmidt, T., et al. (2014). SWISS-MODEL: modelling protein tertiary and quaternary structure using evolutionary information. *Nucleic Acids Research*, *42*(W1), W252–W258. http://doi.org/10.1093/nar/gku340

Beckmann JS and Koppenol WH. (1996) Nitric oxide, superoxide, and peroxynitrite: the good, the bad, and the ugly. *Am J Physiol*. 271:C1424-C1437.

Birke J, Röther W, Jendrossek D (2015) Latex clearing protein (Lcp) of *Streptomyces* sp. strain K30 Is a *b*-Type cytochrome and differs from rubber oxygenase A (RoxA) in its biophysical properties. Appl Environ Microbiol 81:3793–3799. doi: 10.1128/AEM.00275-15

Lyakin, O.Y., Bryliakov, P.K., Britovsek, G.J.P. and Talsi, E.P. (2009) EPR Spectroscopic Trapping of the Active Species of Nonhaem Iron-Catalysed Oxidation. *J Am Chem Soc*. 131, 10798–10799.

Munro OQ and Scheidt WR (1998) (Nitro)Iron(III) Porphyrins. EPR detection of a transient low-spin Iron(III) complex and structural characterization of an O atom transfer product. *Inorg. Chem.* 37:2308-2316.

Nasri HN, Goodwin JA, Scheidt WR (1990). Use of protected binding sites for nitrite binding in Iron(II1) porphyrinates. Crystal structure of the bis(nitro)(a,a,a,a-tetrakis(o-pivalamidophenyl)porphinato)iron(III) anion. *Inorganic Chemistry* 29(2):185-191.

Reynolds M F, Parks R B, Burstyn J N, Shelver D, Thorsteinsson M V, Kerby R L, Roberts G P, Vogel K M and Spiro T G (2000). Electronic absorption, EPR, and Resonance Raman

spectroscopy of CooA, a CO-sensing transcription activator from *R. rubrum*, reveals a five-coordinate NO-haem. *Biochemistry 39*, 388-396

Rodgers KR, Lukat-Rodgers GS, Tang L (2000) Nitrosyl adducts of FixL as probe of haem environment. *J Biol Inorg Chem* 5:642-654. doi: 10.1007/s007750000150

Schmitt G, Seiffert G, Kroneck PMH, Braaz R, Jendrossek D (2010) Spectroscopic properties of rubber oxygenase RoxA from *Xanthomonas* sp., a new type of dihaem dioxygenase. Microbiology (Reading, Engl) 156:2537–2548. doi: 10.1099/mic.0.038992-0

Seidel J, Schmitt G, Hoffmann M, Jendrossek D, Einsle O (2013) Structure of the processive rubber oxygenase RoxA from X*anthomonas* sp. Proc Natl Acad Sci USA 110:13833–13838. doi: 10.1073/pnas.1305560110

Sharma SK, Schaefer AW, Lim H, Matsumura H, Moënne-Loccoz P, Hedman B, Hodgson KO, Solomon EI, Karlin KD (2017) A six-coordinate peroxynitrite low-spin iron(III) porphyrinate complex-The product of the reaction of nitrogen monoxide (·NO(g)) with a ferric-superoxide species. J Am Chem Soc 139:17421–17430. doi: 10.1021/jacs.7b08468
